# Branched poly‐l‐lysine for cartilage penetrating carriers

**DOI:** 10.1002/btm2.10612

**Published:** 2023-10-16

**Authors:** Gavin Gonzales, Jiaul Hoque, Anna Gilpin, Biswanath Maity, Stefan Zauscher, Shyni Varghese

**Affiliations:** ^1^ Department of Biomedical Engineering Duke University Durham North Carolina USA; ^2^ Department of Orthopedic Surgery Duke University School of Medicine Durham North Carolina USA; ^3^ Department of Mechanical Engineering and Materials Science Duke University Durham North Carolina USA

**Keywords:** cartilage penetrating, cationic nanocarrier, intra‐articular administration, osteoarthritis, poly‐l‐lysine

## Abstract

Joint diseases, such as osteoarthritis, often require delivery of drugs to chondrocytes residing within the cartilage. However, intra‐articular delivery of drugs to cartilage remains a challenge due to their rapid clearance within the joint. This problem is further exacerbated by the dense and negatively charged cartilage extracellular matrix (ECM). Cationic nanocarriers that form reversible electrostatic interactions with the anionic ECM can be an effective approach to overcome the electrostatic barrier presented by cartilage tissue. For an effective therapeutic outcome, the nanocarriers need to penetrate, accumulate, and be retained within the cartilage tissue. Nanocarriers that adhere quickly to cartilage tissue after intra‐articular administration, transport through cartilage, and remain within its full thickness are crucial to the therapeutic outcome. To this end, we used ring‐opening polymerization to synthesize branched poly(l‐lysine) (BPL) cationic nanocarriers with varying numbers of poly(lysine) branches, surface charge, and functional groups, while maintaining similar hydrodynamic diameters. Our results show that the multivalent BPL molecules, including those that are highly branched (i.e., generation two), can readily adhere and transport through the full thickness of cartilage, healthy and degenerated, with prolonged intra‐cartilage retention. Intra‐articular injection of the BPL molecules in mouse knee joint explants and rat knee joints showed their localization and retention. In summary, this study describes an approach to design nanocarriers with varying charge and abundant functional groups while maintaining similar hydrodynamic diameters to aid the delivery of macromolecules to negatively charged tissues.

**Translational Impact Statement**

Cationic, branched poly‐l‐lysine nanocarriers that transport through negatively charged tissues could significantly contribute to treating joint diseases promoting deeper penetration into the cartilage, and prolonging drug retention.

## 1 INTRODUCTION

Joint diseases that involve the entire joint, such as osteoarthritis (OA), often require delivery of drugs to chondrocytes residing within the cartilage.^1^, ^2^, ^3^, ^4^ Although intra‐articular drug delivery has been proposed as a more effective strategy compared to systemic administration, the intra‐articular administration of drugs remains a challenge due to their rapid clearance within the joint.^5^, ^6^, ^7^ While nanocarrier‐assisted delivery typically increases the half‐life of therapeutic molecules within the joint, the diffusion of nanocarriers into the cartilage tissue is hindered by its dense and negatively charged extracellular matrix (ECM).^8^, ^9^ Drug delivery is further complicated for treatments that target the whole cartilage and/or subchondral bone that require deeper drug penetration.^3^ Chondrocytes, situated in the lacunae, are sparsely distributed within the anionic ECM, which is comprised of high‐density collagen fibers intertwined with negatively charged proteoglycans.^10^ The challenges associated with delivering drugs to chondrocytes residing within the dense cartilage tissue are considered to be a key bottleneck that contributes to the unsuccessful outcomes of OA treatments.^11^

Cationic nanocarriers that form weak electrostatic interactions with the anionic cartilage ECM can be an effective approach to overcome the electrostatic barrier.^12^, ^13^, ^14^, ^15^, ^16^, ^17^, ^18^ For an effective therapeutic outcome, the nanocarriers first need to adhere to the cartilage tissue, and then penetrate and be retained throughout the full‐thickness cartilage.^19^ It is therefore important to develop nanomaterials that can rapidly adhere upon intra‐articular administration to prevent clearance in the joint and promote transport across the full‐thickness cartilage tissue while maintaining adequate retention within the tissue. Transport of molecules, including nanocarriers, within the cartilage tissue is governed by properties such as size, shape, and surface charge, where the latter strongly influences the cartilage‐binding properties of the nanocarriers and their retention within the tissue.^15^, ^20^ An optimal net positive charge that enables weak, reversible interactions with the cartilage tissue is necessary for the nanocarriers to penetrate through the full‐thickness cartilage tissue, which is ~3 mm for human articular joint cartilage.^21^, ^22^, ^23^ Additionally, the nanocarriers should be able to support high drug loading such that adequate amounts of drug can be delivered with a lower concentration of cationic materials, which are often cytotoxic.^24^ Large amounts of cationic polymers can also alter intra‐tissue osmotic pressure which could have detrimental effects to cartilage tissue.^25^ Research over the years has led to a number of cationic nanocarriers composed of peptides and proteins,^13^, ^14^, ^26^, ^27^ amino acids,^12^ liposomes,^28^ lipid molecules,^29^ or polymers^20^, ^30^, ^31^ to deliver biomolecules to cartilage tissue and also gene editing, with varying levels of success.

Here, we developed branched poly(l‐lysine)‐based cationic nanocarriers of different generations with varying numbers of amine groups and charge density. These branched poly(l‐lysine) (BPL) molecules were designed to have a hydrodynamic diameter of ~40 nm and a dense surface functionality ranging from 30 to 150 primary amines. For example, a second generation (G2) BPL molecule provides ~150 functional amine groups. Employing an *ex vivo* explant culture, we have studied the effect of surface charge and molecular architecture (e.g., number of branches) on BPL uptake, penetration, retention, and desorption into and from cartilage. We have used both healthy and cytokine challenged cartilage explants. Furthermore, we carried out methylprednisolone conjugation to the BPL molecule to examine the possibility of drug conjugation and release. Finally, we examined the *in vivo* retention of BPL molecules using both mouse knee joint explants and a rat knee injury model, which suggest that the molecules localize to the cartilage within 24 h and can be retained within the rat knee joint for at least 14 days.

## 2 RESULTS AND DISCUSSION

### 2.1 Synthesis and characterization of BPL molecules

**FIGURE 1:**
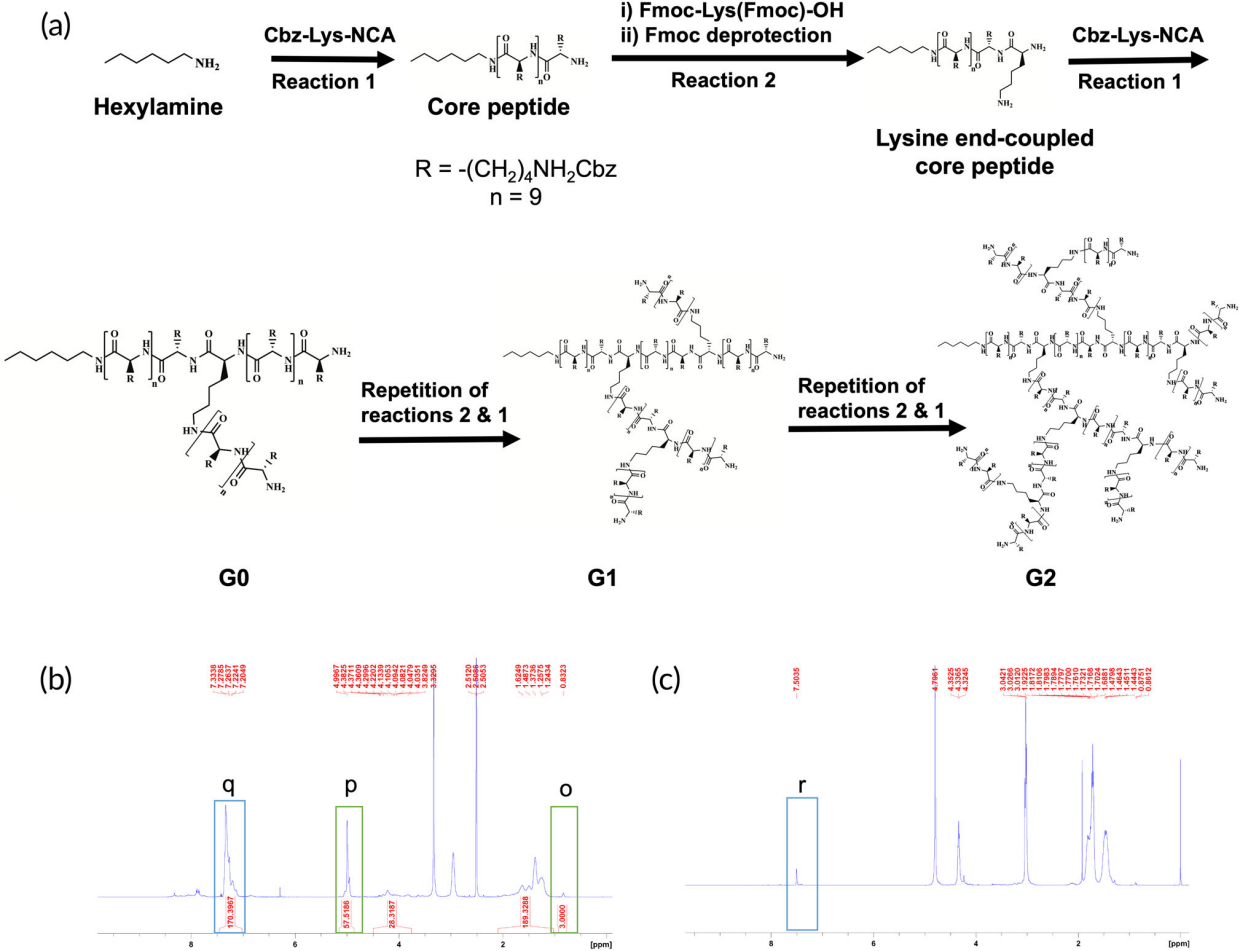
Synthesis and characterization of BPL molecules. (a) Reaction scheme for the synthesis of branched poly (l‐lysine) molecules with varying degrees of branching and functional amine groups. (b) ^1^HNMR spectrum of G0 BPL molecules. The green boxes (region o and p) indicate the presence of the peak from terminal methyl group of hexylamine initiator and ArCH_2_ protons of Cbz‐Lys. The blue box (region q) indicates the presence of the peaks from benzene ring of Cbz group. (c) ^1^HNMR spectrum of completely deprotected G0 BPL molecules. The blue box (region *r*) indicates the absence of the peaks from benzene ring of Cbz group.

The branched poly(l‐lysine) (BPL) molecules were synthesized by ring‐opening polymerization reaction using N^ε^‐benzyloxycarbonyl‐l‐lysine‐N‐carboxyanhydride (Cbz‐Lys‐NCA) as a monomer and an initiator with primary amine group(s) (Figures S1–S3).^32^ First, a lysine oligomer containing 10 lysine units, termed as the core peptide, was synthesized using Cbz‐Lys‐NCA and hexylamine, as described in the Section 3 (Figure S1). Three generations of BPL molecules—generation 0 (G0) with two branches (or arms), generation 1 (G1) with four branches, and generation 2 (G2) with eight branches, were then synthesized by reacting the Cbz‐Lys‐NCA with either the core peptide, G0, or G1 as initiators to generate G0, G1, or G2 BPL molecules, respectively (Figures 1a and S2 and Table S1). Increasing the number of branches provided multivalent BPL nanocarriers with increasing functional groups and charge density without significantly increasing their hydrodynamic diameter. A theoretical calculation, based on the monomer‐to‐initiator feed ratio, suggests that the G0, G1, and G2 BPL molecules possess approximately 30, 70, and 150 functional amine groups, respectively (Table S1).

The successful synthesis of various BPL molecules and their immediate precursors was validated by ^1^HNMR (Figures 1b, c and S4–S11). For instance, Figure 1b, c shows ^1^HNMR spectra of the G0 BPL with and without the benzyloxycarbonyl (Cbz) groups. Comparison of the peak areas at ~0.83 ppm for the terminal –CH_3_ group of the hexylamine unit to that of the aromatic protons of the Cbz groups at ~7.20–7.33 ppm indicated successful polymerization of the lysine monomers (Figure 1b). Furthermore, the ^1^HNMR spectra following the deprotection showed almost complete removal of the Cbz groups as only a negligible signal from the aromatic protons of the Cbz group at 7.20–7.33 ppm could be detected in the NMR spectrum (Figure 1c).^1^HNMR spectra were also used to estimate the average number of lysine units in each branch as well as the total degrees of polymerization (DP) in different generations of the BPL molecules (Table 1). The average DP and chain length estimations based on the ^1^HNMR showed slight deviations from the theoretical predictions, which suggests an incomplete polymerization of BPL molecules at G1 and G2 generations.

**TABLE 1:** Yields and molecular characteristics of branched poly(l‐lysine) molecules of different generations.

Poly(l‐lysine)	Number of branches	Degree of polymerization	Number average molecular weight (from NMR) (g/mol)	Yield (%)	Zeta potential (mV)
Core	0	10	1280	≥90	ND
G0	2	28	3600	≥90	14.9 ± 2.3
G1	4	66	8400	≥80	34.9 ± 1.0
G2	8	146	18,600	≥80	52.0 ± 0.2

Abbreviations: DP, degree of polymerization; ND, not determined.

Dynamic light scattering (DLS) and zeta potential measurements were used to further characterize the BPL molecules. The DLS measurements yielded a mean hydrodynamic diameter of ~40 nm for all BPL molecules (i.e., G0, G1, and G2), with overlapping histograms (Figure S12). We attribute the minimal increase in hydrodynamic diameter, observed as the generation increases, to the intentional branching of the BPL structure. Zeta potential measurements revealed that the surface charge doubled for the BPL molecules from generation G0 to G1, but further increase in branching from G1 to G2 entailed only a slight increase in surface charge (Table 1). We attribute this observation to effective charge screening by the branches at higher BPL generations. Specifically, the G0 BPL molecules had a zeta potential of about 14.9 ± 2.3 mV, which increased to 34.9 ± 1.0 mV for G1 and 52.0 ± 0.2 mV for G2 BPL molecules (Table 1). The increase in surface charge is associated with the increase in the number of functional amine groups from the lysine units presented at the surface of higher generations of BPL molecules.

Since cationic polymers are cytotoxic, we have PEGylated the BPL molecules. Besides reducing cytotoxicity, PEGylation can also be used to vary the surface charge of BPL molecules, which itself could have an effect on their adhesion and penetration into the cartilage tissue. We used two different extents of modification, low PEGylation (LP; ~20% of surface functionalization) and high PEGylation (HP; ~35% of surface functionalization). Details of synthesis and characterization of PEGylated BPL molecules are provided in the Section 3 and Supporting Information (Figures S13–S16). The extent of PEGylation was determined *via* ^1^HNMR spectra by comparing the peaks at 3.62 ppm corresponding to the –OCH_2_CH_2_O– protons of PEG to the peaks at 0.85 ppm corresponding to the terminal –CH_3_ groups of hexylamine initiator (Figures S13–S16). As anticipated, the PEGylation reduced the zeta potential of the BPL molecules, which was determined for G1 and G2 generations. For G1 BPL molecules with ~20% and ~35% PEGylation, the zeta potential values were found to be 24.7 ± 0.9 mV and 25.1 ± 0.8 mV, respectively. A similar trend was also observed with G2 BPL molecules, where the zeta potential has decreased to 42.5 ± 0.2 mV and 40.6 ± 0.9 mV with ~20% and ~35% PEGylation, respectively. The PEGylation reduced the surface charge of the G1 and G2 BPL molecules as some of the free amine groups of the lysine units were utilized for the chemical conjugation of PEG molecules.

### 2.2 Cartilage uptake, penetration, and retention of BPL molecules

**FIGURE 2:**
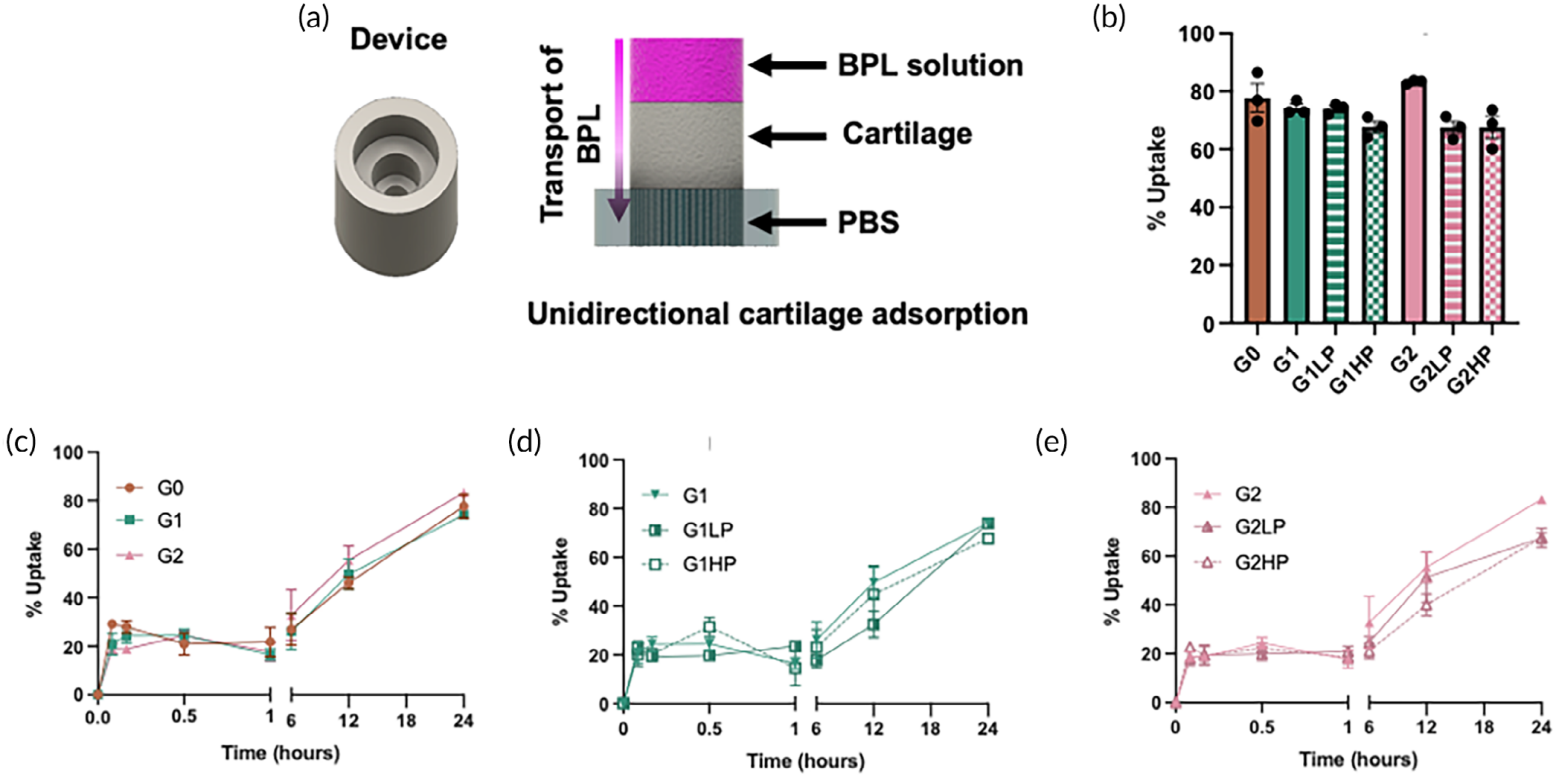
Uptake of BPL molecules by cartilage explants. (a) Schematic illustration of the BPL uptake experiment using cartilage explants. (b) The uptake percentage of BPL molecules in cartilage explants after 24 h, and (c–e) the uptake of various BPL molecules as a function of time up to 24 h. The statistical analysis of (b) is provided in Table S2.

The adhesion and transport of BPL molecules across the cartilage explants were assessed using a custom‐designed device that allowed selective exposure of the BPL solution to the superficial zone of the cartilage explant (~4 mm thickness) and allowed diffusion only in one direction (i.e., from top to bottom) (Figure 2a). Cartilage uptake of different generations of BPL molecules tagged with cyanine 5 (Cy5) were determined by measuring relative changes in the fluorescence intensity of the solution (i.e., depletion of BPL molecules in the solution) as a function of time. The cartilage uptake and depth of penetration was further confirmed by image analysis of the cartilage explants. These experiments revealed a rapid initial uptake in the first 5 min, followed by a steady plateau for about 60 min. A further continuous increase in uptake was observed from about 2 to 6 h. By 24 h, ~ 80% of the BPL molecules were taken up by the cartilage explant in all different generations of BPL molecules (Figure 2b). While all the different BPL molecules showed high affinity for cartilage, there were notable differences in the amount taken up within the first 5 min of exposure. Specifically, G0 BPL molecules showed a slightly higher uptake (29 ± 2%) compared to G1 and G2 generations, which showed similar levels of initial uptake, 21 ± 4% for G1 and 19 ± 2% for G2 within the first 5 min (Figure 2c). We attribute the higher initial uptake of the G0 BPL molecules to their lower surface charge and slightly smaller size, both of which facilitate faster transport into the cartilage, compared to G1 and G2 BPL molecules. Interestingly, PEGylation of G1 and G2 BPL molecules did not significantly affect their uptake by cartilage (Figure 2b,d,e), which suggests that the PEGylated BPL molecules possess the optimal surface charge required for cartilage adhesion and penetration. These observations are consistent with previous studies which suggested that the transport of cationic nanocarriers across the cartilage tissue is associated with an optimal surface net charge.^11^, ^15^, ^20^, ^24^, ^33^

**FIGURE 3:**
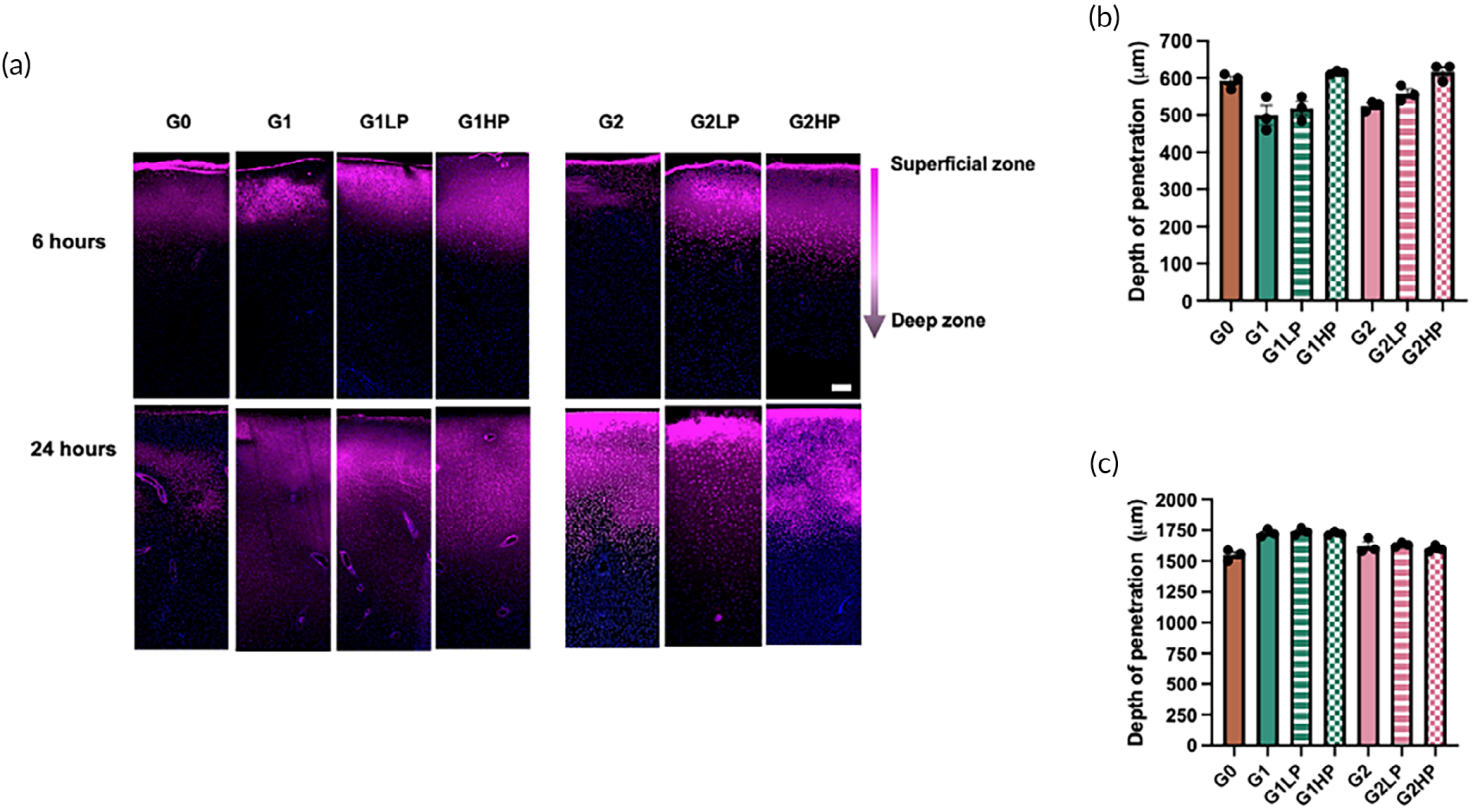
Penetration of BPL molecules within the cartilage explant. (a) Fluorescence images showing intra‐cartilage penetration of various BPL molecules from superficial zone to deep zone (indicated by the arrow) after 6 and 24 h. Scale bar: 200 μm. (b, c) Quantification of the depth of penetration of different BPL molecules at 6 and 24 h. The statistical analyses for (b) and (c) are provided in Tables S3 and S4, respectively.

To characterize the depth of penetration, the cartilage explants were removed from the device after 6 and 24 h of exposure to different BPL molecules, sectioned and imaged (Figure 3a). We found that all the BPL molecules penetrated the cartilage explant by 6 h of exposure (Figure 3a). However, G0 BPL molecules penetrated deeper (590 ± 30 μm) compared to G1 and G2 BPL molecules, which penetrated 500 ± 50 μm and 520 ± 25 μm, respectively (Figure 3b). We attribute the deeper penetration by the G0 BPL molecules to their higher mobility within the cartilage tissue, aided by their lower surface charge, compared to G1 and G2 BPL molecules. These findings are consistent with the results from our cartilage uptake measurements (Figure 2b–e). The penetration depth, measured after 6 h of incubation time, increased with increasing PEGylation and was similar to the penetration of G0 BPL molecules (Figure 3b). These observations further underscore the importance of weak interactions between the BPL molecules and cartilage in promoting their transport across a thick cartilage tissue. Fluorescent images in Figure 3a show that after 24 h of exposure, the BPL molecules penetrated further into the cartilage tissue; that is, we found a penetration depth for G0, G1, and G2 BPL molecules of 1550 ± 50 μm, 1710 ± 30 μm, and 1620 ± 25 μm, respectively (Figure 3c). These results suggest that despite the changes in molecular architecture (i.e., branching, PEGylation) and surface charge, all of the different BPL molecules significantly penetrated into the cartilage explant by 24 h; that is, to depths that are on the same order of magnitude as human cartilage tissue thickness.^21^, ^22^, ^23^

**FIGURE 4:**
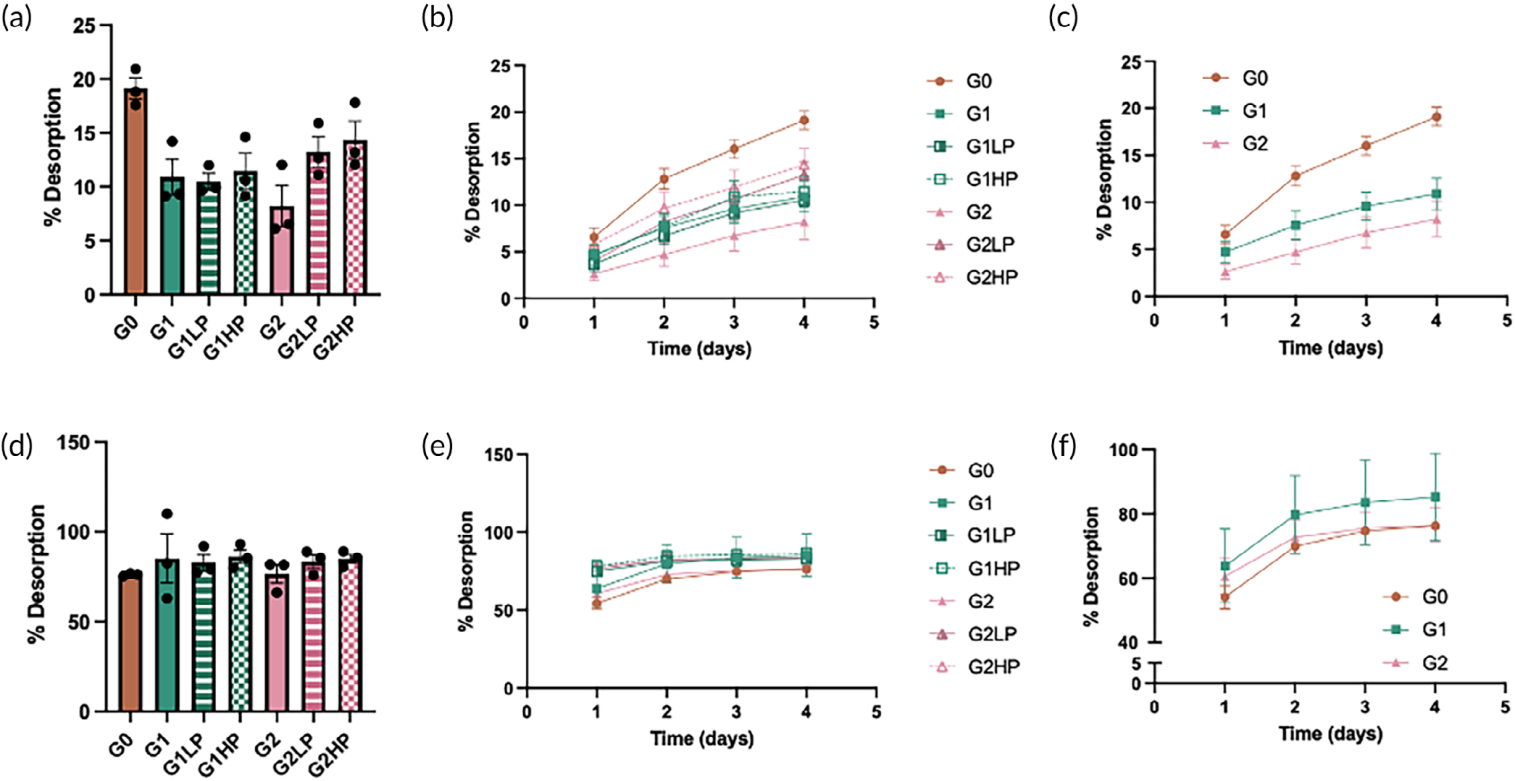
Desorption of BPL molecules from the cartilage explant. (a, d) Desorption of BPL molecules from the cartilage explants after 4 days in 1× PBS or 10× PBS at 37°C. (b, e) The BPL concentration in 1× and 10× PBS as a function of time. (c, f) Comparison of the desorption of G0, G1, and G2 BPL molecules in 1× and 10× PBS as a function of time. The statistical analyses for (a) and (d) are provided in Tables S5 and S6, respectively.

To further characterize the electrostatic interactions between the negatively charged cartilage ECM and the positively charged BPL molecules, we performed a desorption assay by incubating the BPL‐absorbed cartilage explants in 1× or 10× phosphate buffered saline (PBS) and measuring the amount of BPL molecules released into the solution as a function of time for up to 4 days. The results showed that around ~20% of G0 BPL molecules and 10%–15% of all other BPL molecules, including the PEGylated G1 and G2 molecules, were desorbed from the cartilage explants in 1× PBS after 4 days (Figure 4a–c). The effect of branching (G0, G1, and G2) and PEGylation on desorption is shown in Figures 4c and S17a,b, respectively. In contrast, nearly all BPL molecules were desorbed (75%–95%) from the cartilage explants in 10× PBS, a solution with higher ionic strength (Figures 4d–f and S17c,d). We also observed that PEGylation of G2 BPL molecules promoted their desorption in 1× PBS compared to the non‐PEGylated G2 BPL molecules (Figure S17b). The higher desorption of G0 BPL and PEGylated G2 BPL molecules in 1× PBS could be attributed to the reduced surface charge along with the likely screening effect from the PEG chains, resulting in weaker electrostatic interactions with the negatively charged cartilage matrix.

By adapting an established protocol, we further examined the binding properties of the various BPL molecules with the cartilage matrix.^12^, ^15^, ^34^ An equilibrium uptake assay was performed by incubating a thin cartilage tissue with varying concentrations of BPL molecules (*C*_initial_) followed by measuring the fluorescence intensity after 24 h of incubation (*C*_final_). We found that with increasing initial BPL concentration (*C*_initial_), the uptake ratio of the Cy5‐labeled BPL molecules also increased; a representative uptake curve for G0 BPL is shown in Figure S18a. The cartilage uptake equilibrium (*R*_*u*_) of the BPL molecules was calculated and correlated to *C*_final_ as described in the Section 3 (Figure S18b). The estimated reversible binding properties of the BPL molecules with the cartilage tissue is given in Table S7. The partition coefficient (K) for BPL molecules decreased with increasing surface charge, and was ~2.8, ~1.5, and ~ 1.1 for G0, G1, and G2, respectively. The binding site concentration (N) of the BPL molecules showed a similar trend with G0, G1 and G2 having ~708, ~284, and ~77 μM, respectively. The values for the equilibrium dissociation constant K_D_ for G0, G1, and G2 were ~221, ~1301, and ~6574 μM, respectively, which indicates an increase in K_D_ with increasing surface charge. These trends further suggest the importance of surface charge of the BPL molecules on cartilage binding and transport. PEGylation was found to increase the equilibrium partition coefficient (K) and the binding site density (*N*) for the G2HP molecules to ~2.2 and ~384 μM, respectively. The equilibrium dissociation constant (*K_D_*) of the PEGylated G2 BPL molecules decreased, and it is estimated to be ~247 μM for G2HP. The observed decrease with PEGylation could be due to their weaker reversible electrostatic interactions with the cartilage tissue. More binding sites and reversible binding to cartilage ECM is necessary to maintain an enhanced intra‐tissue concentration for sustained delivery. The reversible binding also ensures the effective transport of the nanocarriers across a full‐thickness cartilage tissue.

### 2.3 Cytotoxicity of BPL molecules

**FIGURE 5:**
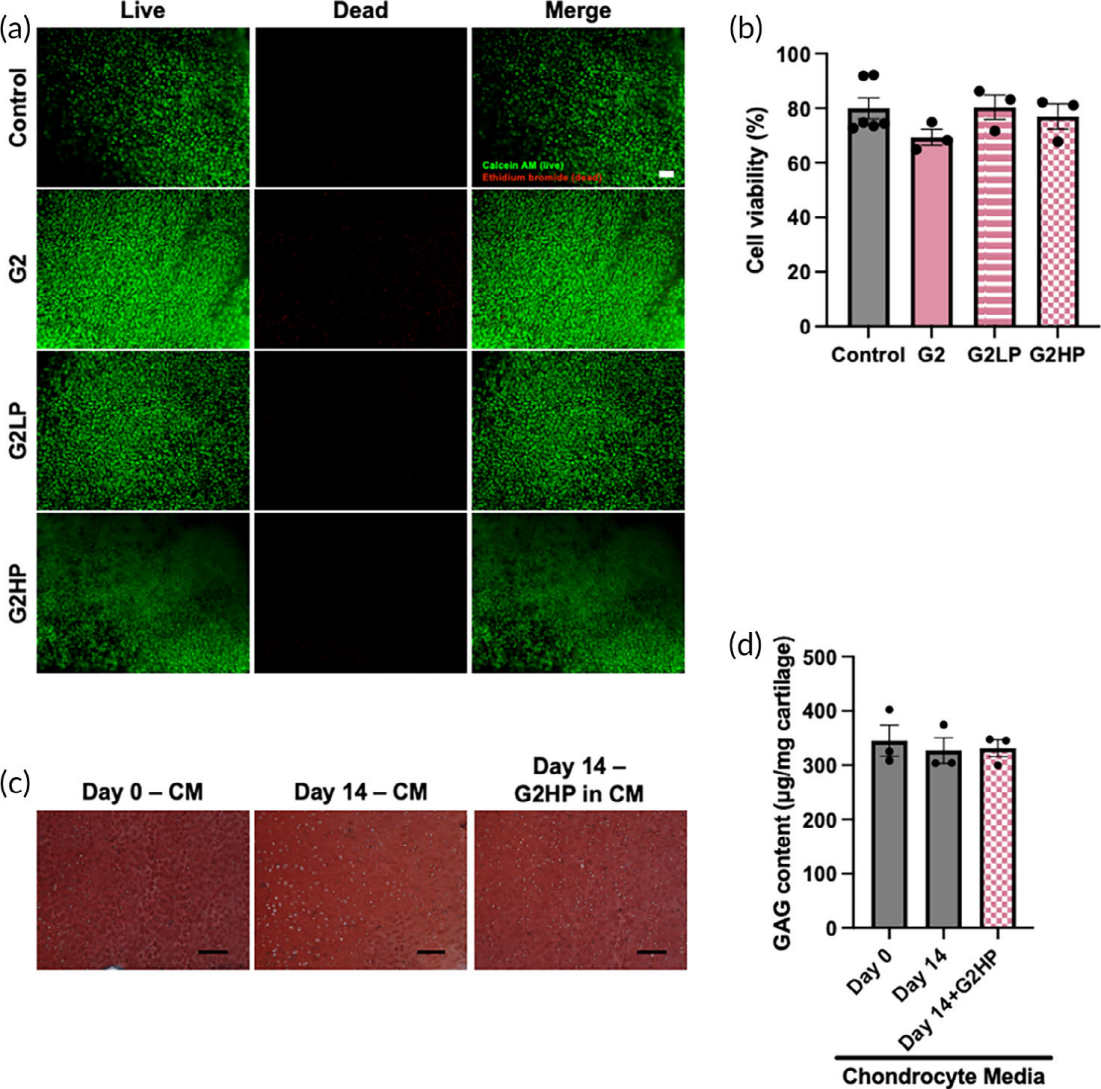
Chondrocyte toxicity of BPL molecules. (a) Fluorescence images of the explant following 24 h of culture in chondrocyte medium supplemented with 11.5 μM of BPL molecules. Green: live cells. Red: dead cells. Scale bar: 100 μm. (b) Quantification of cell viability from the live/dead images. (c) Safranin‐O stained cartilage explants cultured in chondrocyte media (CM) with or without G2HP BPL molecules at a concentration of 11.5 μM. Scale bar: 100 μm. (d) Glycosaminoglycan (GAG) content of the cartilage explants. The statistical analyses for (b) and (d) are provided in Tables S8 and S9, respectively.

To determine the cytotoxicity of BPL molecules, cartilage explants were incubated with various BPL molecules at two different concentrations, ~11.5 and 23 μM, for 24 h and analyzed for live and dead cells (Figures 5a,b and S19).^35^ While both the concentrations of non‐PEGylated (G0, G1 and G2) BPL molecules showed some level of toxicity, cartilage explants exposed to 23 μM BPL showed higher cell death (Figure S19). The cytotoxicity was more prominent with G2 BPL molecules likely because of the higher positive charge (Figures 5a,b and S19). Cationic biomaterials including nanocarriers are known to be cytotoxic and the toxicity is dependent on their positive surface charges.^12^, ^36^ PEGylation is widely used to reduce the cytotoxicity where it provides a screening effect to the positively charged cationic molecules, and thus reducing the overall surface charge. In agreement with this widely adapted strategy, PEGylated G2 BPL molecules showed less toxicity compared to the bare G2 BPL molecules. All additional characterization involving cartilage tissues and the *in vivo* studies were carried out using either G2 and/or G2HP BPL molecules.

Histological and biochemical analyses for sulfated proteoglycans were carried out to further determine the effect of the BPL molecules on the biosynthetic activity of the cartilage explants cultured in chondrocyte medium supplemented with G2HP BPL molecules for 14 days.^37^, ^38^ Safranin‐O staining of the cartilage explants showed similar levels of staining among all the groups, as well as explants at the initial time point (Figure 5c). Additionally, biochemical analyses for the proteoglycan content of the explants from various experimental groups showed similar levels of glycosaminoglycan (GAG) content. Specifically, explants cultured in medium with and without G2HP BPL molecules had a GAG content of 331 ± 16 and 327 ± 24 μg/mg, respectively, which is similar to the value for cartilage explants at the initial time point 345 ± 25 μg/mg (Figure 5d). Together these results suggest minimal‐to‐no detrimental effect of G2HP BPL molecules on chondrocyte viability and function.

We also examined the penetration of G2 and G2HP BPL molecules across an IL‐1β‐challenged cartilage explant as a function of time. Exposure to IL‐1β is widely used to mimic the proteoglycan loss associated with early stages of OA.^39^ The IL‐1β‐conditioned cartilage explants exhibited higher uptake of G2 and G2HP BPL molecules compared to healthy cartilage, with approximately 20% higher uptake occurring after 6 h (Figure S20a). However, there was no such change in BPL uptake among the healthy and IL‐1β challenged cartilage explants at 24 h (Figure S20b). The BPL molecules also showed higher penetration depth in IL‐1β conditioned explants compared to the healthy explants (Figure S20d–g). There was no significant difference in the uptake or depth of penetration between the G2 and G2HP BPL molecules (Figure S20c,f). Since synovial fluid can influence the function of nanocarrier by inducing their aggregation, we also examined the behavior of G2 and G2HP BPL molecules in presence of synovial fluid.^40^ Samples containing G2 BPL molecules showed macroscopic aggregation of the nanocarriers in the presence of synovial fluid and precipitated out of the solution while no such phase separation/aggregation was observed in any of the samples containing G2HP BPL molecules (Figure S21).

### 2.4 Joint retention of G2HP BPL molecules

**FIGURE 6:**
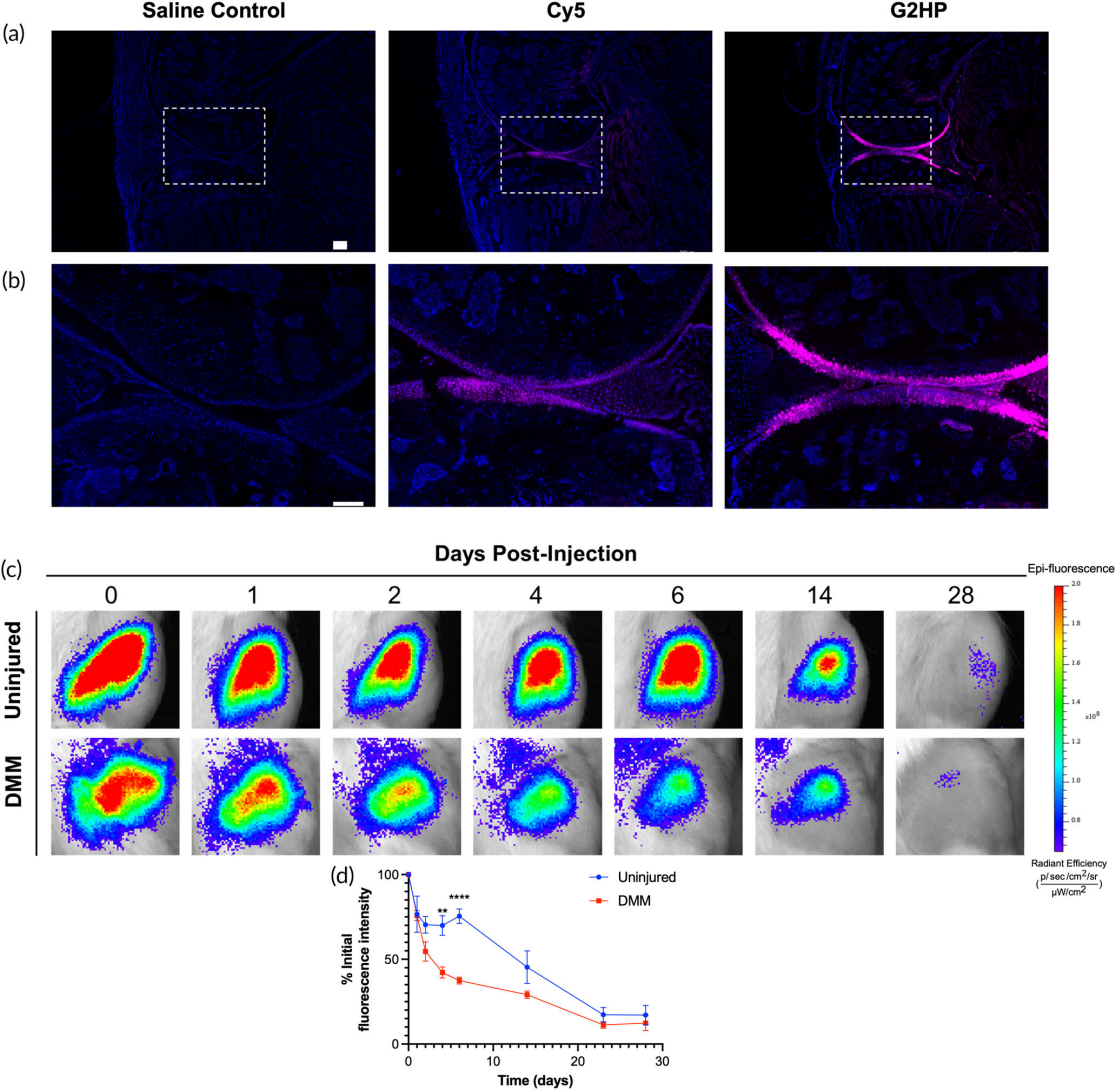
PEGylated BPL molecules show joint residence and cartilage penetration in rodents. (a, b) Fluorescence images of the mouse knee joint following intra‐articular injection of Cy5‐G2HP BPL molecules with magnified images shown in (b). Scale bars: 200 μm. (c) Representative IVIS images of the rat knee joints over 28 days following intra‐articular injection of Cy7‐conjugated G2HP BPL molecules. (d) Time course of fluorescent radiant efficiency of the BPL within the joints. Data are means ± 95% CIs of nonlinear fit, *n* = 4 joints per formulation. The statistical analysis for (d) is provided in Table S16.

To further assess penetration of the BPL molecules into the cartilage following their intra‐articular injection, we examined the distribution of the injected Cy5‐labeled G2HP BPL within a mouse knee joint explant. Following 24 h of injection, the mouse knee joint explants were sectioned and imaged *via* fluorescence microscopy. The fluorescence images showed the presence of Cy5‐labelled G2HP BPL molecules throughout the cartilage (Figure 6a, b). Joints injected with the same volume of Cy5 showed minimal to no Cy5 signal at the same exposure time.

We also examined the retention of the G2HP BPL molecules as a function of time following their intra‐articular administration to rat knee joints by using Cy7‐conjugated BPL molecules (Cy7‐G2HP).^41^ To address the effect of joint injury on BPL retention, a surgical destabilization of the medial meniscus (DMM) model was used and compared against the uninjured knee joint. One week following the surgical procedure, the Cy7‐G2HP was administered into the joint by intra‐articular injection. Both the uninjured and injured joints that received Cy7‐G2HP were monitored for 28 days using IVIS imaging. The fluorescent signal of Cy7‐G2HP BPL molecules was detected for at least 14 days in both knee joints, with the signal being about 50% of the initial fluorescence intensity (Figure 6c). Residual signal was detected in both the uninjured and injured joints until 28 days (maximum experimental time used). The fluorescent intensity of the region of interest, shown in Figure 6d, suggests that the Cy7‐G2HP BPL molecules' signal attenuated at a slower rate in the uninjured knee joint than in the injured joint. Further analysis of the data indicated that the half‐life of Cy7‐G2HP BPL molecules was 12 and 3.4 days in uninjured and injured knee joints, respectively. The lower retention of the BPL molecules in the injured knee could be attributed to the increased inflammation or the changes caused by surgery leading to a faster clearance from the knee joint. Together, the results demonstrate that G2HP BPL molecules can be retained in the knee joint for days to weeks while molecules with similar molecular weight are reported to be cleared in hours.^42^, ^43^

### 2.5 G2HP BPL molecules as nanocarriers for methylprednisolone

**FIGURE 7:**
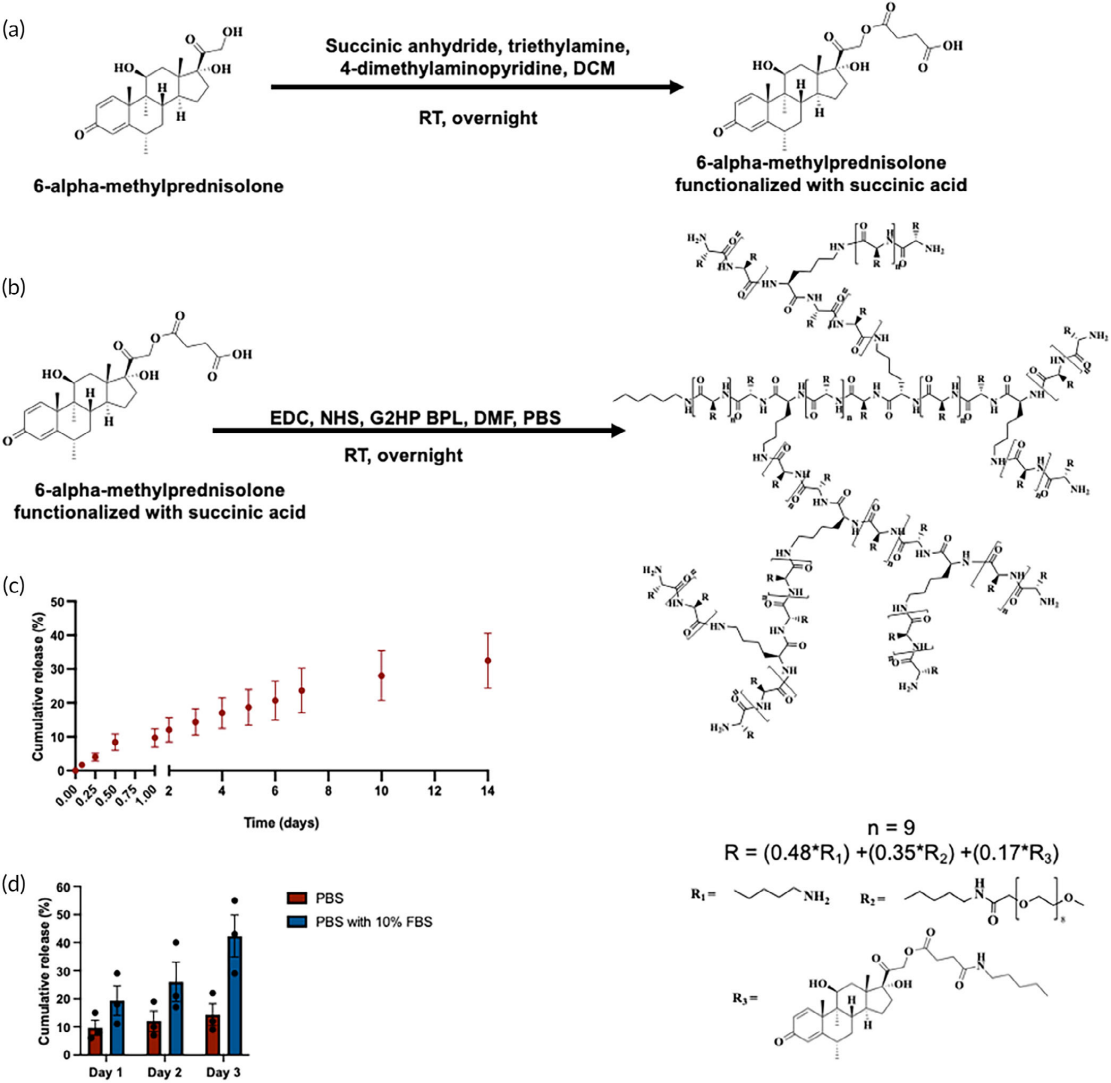
Conjugation and release of methylprednisolone. (a, b) Reaction scheme showing functionalization 6‐alpha‐methylprednisolone with succinic acid and its conjugation to the G2HP BPL molecules. (c) Release of methylprednisolone from the G2HP BPL molecules in PBS (d) Release of methylprednisolone from the G2HP BPL molecules in PBS and PBS with 10% FBS. The statistical analysis for (d) is provided in Tables S17 and S18.

To further validate the potential of the BPL molecules to be used as a drug carrier, we conjugated methylprednisolone, a steroid which is used to treat osteoarthritis,^44^ to the G2HP BPL molecules using an amide coupling reaction as shown in Figure 7a, b. Towards this, methylprednisolone was functionalized with succinic acid and then reacted with BPL molecules containing amine functional group. The successful conjugation of the methylprednisolone to the BPL molecules was validated with NMR and the methylprednisolone incorporation to the G2HP BPL molecules was calculated to be ~17% of the total mass (Figures S22–S25). Methylprednisolone release from the BPL molecules into PBS as a function of time was evaluated over a period of 14 days.^45^ The release profile was steady and sustained. After 1 day, ~9.3% of the methylprednisolone was released in PBS, and this value was increased to ~14.3% by day 3, and by 14 days, 32.4% of the drug was released from the BPL molecules (Figure 7c). Since the drug was conjugated *via* an ester bond, we also examined the drug release in PBS containing 10% FBS, as FBS contains esterase, which can be a trigger for the methylprednisolone release. As anticipated, we observed a significantly higher amount of methylprednisolone release from the BPL molecules was observed in presence of FBS; by day 3 (maximum experimental time measured) ~42.3% of methylprednisolone was released from the carrier (Figure 7d).

## 3 MATERIALS AND METHODS

### 3.1 Synthesis of the BPL molecules

The branched poly(l‐lysine) (BPL) molecules were synthesized *via* ring‐opening polymerization of N^ε^‐benzyloxycarbonyl‐l‐lysine‐N‐carboxyanhydride (Cbz‐Lys‐NCA) following a series of reactions as shown in the reaction scheme (Figures 1a and S1–S3).^32^ Synthesis of each compound/intermediate product are described in detail below.

#### 3.1.1 Synthesis of N^ε^‐benzyloxycarbonyl‐ l ‐lysine‐N‐carboxyanhydride (Cbz‐Lys‐NCA)

Cbz‐Lys‐NCA was synthesized by reacting N^ε^‐benzyloxycarbonyl‐l‐lysine (Cbz‐Lys) (Cat. no. 96840; Sigma‐Aldrich) with triphosgene (Cat. no. T1467; TCI Chemicals) at 50°C in THF (Cat. no. 186562; Sigma‐Aldrich) for 3 h (Figure S1a). The reaction mixture was precipitated and washed in hexane (Cat. no. 197360025; Acros Organics) and vacuum dried overnight prior to the following experiments.

#### 3.1.2 Synthesis of oligo(Cbz‐ l‐lysine) core peptide

A solution of Cbz‐Lys‐NCA in dry DMF (0.2 g/ml; Cat. no. 9227056; Sigma‐Aldrich) was introduced into a Schlenk flask fitted with a drying tube. To this, n‐hexylamine (Cat. no. 219703; Sigma‐Aldrich) (1:10::n‐hexylamine: Cbz‐Lys‐NCA) was added and the reaction was carried out at room temperature for 5 days under stirring (Figure S1b). The reaction mixture was slowly added to a ~ 20‐fold excess of water, and the precipitated product (termed as core peptide hereafter) was filtered using a Büchner funnel fitted with Whatman grade 1 filter paper (Cat. no. 1001‐070; Whatman), washed with DI water, flash frozen in liquid nitrogen, and freeze dried. The core peptide was subsequently reacted with N^α^,N^ε^‐di(9‐fluorenylmethoxycarbonyl L‐lysine) (N^α^,N^ε^‐diFmoc; Cat. no. 8520410025; Sigma‐Aldrich).

#### 3.1.3 N^α^,N^ε^‐diFmoc coupling to the core peptide

A round‐bottom flask containing the core peptide in DMF was reacted with four equivalents of N^α^,N^ε^‐diFmoc, four equivalents of 2‐(1H‐benzo‐triazole‐1‐yl)‐oxy‐1,1,3,3‐tetramethyluronium hexafluorophosphate (HBTU; Cat. no. 8510060025; Sigma‐Aldrich), and 12 equivalents of 1‐hydroxybenzotriazole (HOBt; Cat. no. 157260; Sigma‐Aldrich). To this, 10 equivalents of N,N‐diisopropylethylamine (DIPEA; Cat. no. 496219; Sigma‐Aldrich) were added and the reaction was carried out at room temperature for 3 days (Figure S1c). The reaction mixture was precipitated in water, flash frozen in liquid nitrogen, and freeze‐dried. The resulting reaction product was washed several times with diethyl ether (Cat. no. 32203; Sigma‐Aldrich), vacuum dried, and used for the following reaction.

#### 3.1.4 Selective removal of Fmoc‐protected groups of the end‐functionalized polypeptides

A solution of N^α^,N^ε^‐diFmoc‐Lys end‐functionalized oligopeptide in DMF was reacted with piperidine (~ 20% (v/v)) (Cat. no. 104094; Sigma‐Aldrich) in a round bottom flask at room temperature for 1 h and precipitated in water (Figure S1d). The precipitated poly(l‐lysine) was flash frozen in liquid nitrogen and freeze‐dried. The freeze‐dried product was washed several times with diethyl ether, and vacuum dried.

#### 3.1.5 Generation of branched poly(l‐lysine) (G0)

The poly(l‐lysine) molecules with active amine functional groups, generated by the removal of Fmoc groups, were dissolved in DMF and reacted with Cbz‐Lys‐NCA (1:10::terminal amine group: Cbz‐Lys‐NCA) (Figure S1e). After 5 days, the solution was slowly added to a 20‐fold excess of water. The precipitated polymers were filtered using a Büchner funnel fitted with Whatman grade 1 filter paper, flash frozen in liquid nitrogen, and freeze‐dried. This reaction results in a branched poly(l‐lysine) containing ~30 lysine units.

#### 3.1.6 Generation of G1 and G2 BPL molecules

The above reaction steps were repeated to synthesize lysine oligomers with higher branched structures to achieve next generation of BPL molecules (G1 and G2) (Figure S2). Following reaction 3, reactions 2i, 2ii, and 1 in Figures S1 and S2 were repeated in order to add more lysine units to the branched structures. Briefly, an Fmoc‐protected lysine was conjugated to the terminal amine group of G0 BPL molecules, and subsequently deprotected to obtain two free amine groups at the end of each arm of G0. Then, as in reactions 1 and 3, lysine end‐functionalized G0 peptide and Cbz‐Lys‐NCA monomer (1:10::terminal amine group: Cbz‐Lys‐NCA) were reacted in DMF for 5 days at 37°C. The reaction mixture was then slowly added to a 20‐fold excess of water to precipitate. The precipitate was filtered using a Büchner funnel fitted with a Whatman grade 1 filter paper, flash frozen in liquid nitrogen, and freeze‐dried to obtain G1 BPL molecules. G2 BPL molecules was similarly generated by repeating the above reaction steps using G1 BPL as the starting material and Cbz‐Lys‐NCA as the monomer.

#### 3.1.7 Complete deprotection of BPL molecules

A round‐bottom flask with a solution of the branched poly(l‐lysine) in trifluoroacetic acid (TFA, ~100 mg/3 ml) (Cat. no. A12198‐36; Alfa Aesar) was reacted with a 4‐fold molar excess of a 33 wt. % hydrobromic acid (Cat. no. 18735; Sigma‐Aldrich) in acetic acid (Cat. no. 695092; Sigma‐Aldrich) for 1 h at room temperature (Figure S3). The reaction mixture was precipitated in diethyl ether and the product in quantitative yield after filtration and vacuum‐drying was stored at −20°C.

#### 3.1.8 Functionalization of BPL molecules with poly(ethylene glycol)

The BPL molecules were PEGylated by reacting with N‐hydroxysuccinimide (NHS) methyl‐PEG8 (mPEG8‐SCM; Cat. no. PJK‐209; Creative PEGworks) (Figure S26). Briefly, BPL molecules were dissolved in 1× PBS and reacted with a solution of mPEG8‐SCM in DMSO (Cat. no. 276855; Sigma‐Aldrich) for 4 h at room temperature. The solutions were dialyzed for 3 days using Spectra/Por 3 Dialysis Tubing with a MWCO 3.5 kDa (Cat. no. 132724; Repligen) in DI water, flash frozen in liquid nitrogen, and freeze‐dried.

#### 3.1.9 Functionalization of BPL molecules with cyanine dyes

The cyanine dyes (Cy5 or Cy7) were conjugated to BPL molecules by reacting with N‐hydroxysuccinimide (NHS) Cy5 or Cy7 esters (NHS‐Cy5 or NHS‐Cy7; Cat. no. 43310 and 45020; Lumiprobe). Briefly, BPL molecules were dissolved in 1× PBS and reacted with a solution of NHS‐Cy5 or NHS‐Cy7 in DMSO for 12 h at room temperature. The solutions were dialyzed for 5 days using Spectra/Por 3 Dialysis Tubing, 3.5 kDa MWCO, flash frozen in liquid nitrogen, and freeze‐dried.

#### 3.1.10 Conjugation of succinic acid linker to 6‐alpha‐methylprednisolone

To a suspension of 6‐alpha‐methylprednisolone (1.38 mmol, Cat. no. 338800050; Acros Organics) in dry DCM, (2 mg/ml, Cat. no. 270997; Sigma Aldrich), succinic anhydride (2.67 mmol, Cat. no. 239690; Sigma Aldrich) and triethylamine (Cat. no. T0886; Sigma Aldrich) were added under stirring.^46^ The mixture was stirred for 10 min and the catalytic agent 4‐dimethyaminopyridine (0.1 mmol, DMAP, Cat. no. 107700; Sigma Aldrich) was added to it. The reaction was then continued overnight at room temperature. The reaction mixture was precipitated in 1 N HCl and solid precipitate was filtered, washed with water, and vacuum dried to get the white powder of 6‐alpha‐methylprednisolone conjugated with succinic acid in 85% yield.

#### 3.1.11 Conjugation of the modified methylprednisolone to G2HP BPL molecules

The methylprednisolone functionalized with succinic acid was conjugated into the BPL molecules by using amide coupling method. Briefly, the methylprednisolone functionalized with succinic acid (0.084 mmol), 1‐ethyl‐3‐(3‐dimethylaminopropyl)carbodiimide (EDC; 0.34 mmol, Cat. no. D1601; TCI Chemicals), and NHS (0.34 mmol, Cat. no. 130672; Sigma Aldrich) were dissolved in dry DMF and stirred overnight. The reaction mixture was transferred into a solution of G2HP BPL molecules (0.5 μmol) dissolved in PBS and further reacted for 24 h. The reaction mixture was placed in a 3.5‐kDa membrane and dialyzed against 10% DMSO/water for 24 h, followed by dialysis in DI water for 2 days. The reaction mixture was flash frozen in liquid nitrogen and freeze dried to get the solid product, methylprednisolone conjugated to G2HP BPL. Successful conjugation of 6‐methylprednisolone was determined by^1^HNMR analysis.

### 3.2 Characterization of BPL

#### 3.2.1 Proton nuclear magnetic resonance spectroscopy ( ^1^HNMR)

Successful synthesis of BPL molecules was characterized by^1^HNMR. For^1^HNMR measurements, the products were dissolved in deuterated water (Cat. no. 151882; Sigma Aldrich), dimethyl sulfoxide‐d6 (Cat. no. 364650500; Acros Organic) or methanol‐d_4_ (Cat. no. 343803; Sigma Aldrich) at a concentration of ~1 wt. %. The spectra were recorded with a 500 MHz Agilent/ Varian VNMRS spectrometer at room temperature.

#### 3.2.2 Zeta potential characterization

The BPL molecules were dissolved in DI water at concentrations of 3.5–7.5 μM, filtered through a 0.22 μm filter (Cat. no. 431229; Corning) and used for zeta potential measurements. A Malvern Zetasizer Nano Series was used with disposable folded capillary cells (Cat. no. DTS1070; Malvern Panalytical) for the measurements. The zeta potential value was determined by averaging three independent measurements, where each measurements involved 100 runs, and the Smoluchowski method was used to calculate the zeta potential value.

#### 3.2.3 Dynamic light scattering measurements

For dynamic light scattering (DLS) measurements, the polymers were dissolved at 125 μg/ml in PBS and filtered through a 0.22 μm filter. A DynaPro Plate Reader was used with black Corning 96‐well plate with clear bottom (Cat. no. CLS3631; Sigma‐Aldrich) for DLS measurements.

#### 3.2.4 Aggregation of G2 and G2HP BPL molecules in synovial fluid

The G2 and G2HP molecules were dissolved at 625 μg/ml in PBS, and to this bovine synovial fluid (Cat. no. BOV00SYNFL‐0110555; BioIVT) was added until the G2 and G2HP molecules were at a concentration of 500 μg/ml, and the synovial fluid was at a concentration of 20% (v/v).^40^ After the solutions were made, the BPL and synovial fluid solutions were placed in an incubator at 37°C, shaking at 100 rpm for 30 min. After the 30‐min incubation, digital images were taken of the solutions. The aggregates were also imaged with a microscope.

#### 3.2.5 Bovine cartilage explants

Two‐week‐old bovine knee joints were obtained from Research 87 (Boylston, MA), and cartilage discs of ~4 mm diameter and thickness were harvested from the femur and stored in chondrocyte media DMEM (Cat. no. 11965092; Gibco), 10% (v/v) FBS (Cat. no. 16000044; Gibco), 1% (v/v) ascorbic acid (Cat. no. A4544; Sigma‐Aldrich), 1% (v/v) HEPES (Cat. No. 15630080; Gibco), 1% (v/v) L‐proline (Cat. no. P5607; Sigma‐Aldrich), 1% (v/v) sodium pyruvate (Cat. no. 11360070; Gibco), 1% (v/v) MEM NEAA (Cat. no. 11140050; Gibco) and 1% (v/v) penicillin–streptomycin (Cat. no. 15140122; Gibco) at 37°C in an incubator (~24 h) prior to use.

#### 3.2.6 Adhesion and penetration of the BPL molecules across cartilage explants

Bovine cartilage explants of 4 mm in diameter and 4 mm thickness were equilibrated in chondrocyte media 24 h prior to use. A custom designed device that enables selective exposure of the superficial zone of the cartilage to the BPL solution was used for the experiments. The device (Figure 2a) was generated from polydimethylsiloxane (PDMS; Ellsworth Adhesives) and the master molds with the required features were 3D printed using polylactic acid (PLA) filaments. Specifically, the PLA master mold was designed to create a cylindrical cartilage holder with an outer diameter of 12 and 15 mm thick and an inner compartment of three segments of equal length (5 mm) but varying diameters (upper diameter: 10 mm, middle diameter: 3.95 mm, and lower diameter: 2 mm). Cartilage explants were press fitted into the middle chamber of the PDMS mold and exposed to the upper compartment, which was designed to accommodate 200 μl of BPL solutions. The lower compartment was used to access the PBS when the device was placed in a 24‐well plate containing 500 μl of PBS to maintain humidity and limit dehydration of the cartilage samples.

Cyanine 5 (Cy5)‐conjugated BPL (Cy5‐BPL) molecules were dissolved in 1× PBS at a concentration of 500 μg/ml and used for the experiments. The superficial zone of cartilage explants was exposed to 200 μl of the Cy5‐BPL solution and incubated at 37°C. Adhesion and penetration of BPL molecules to the cartilage explants was determined as a function of time (0–24 h). The BPL solutions were collected at predetermined time intervals, and fluorescence intensities were measured with excitation and emission wavelengths of 515 and 580 nm, respectively. The difference between the fluorescence intensities of the Cy5‐BPL molecules before and after incubation was used to determine the amount of BPL molecule taken up by the explant. The concentration of BPL molecules in the solution was estimated from a standard curve and the % uptake of the BPL molecules was calculated: percent of BPL uptake = {(BPL uptake/Initial BPL amount) × 100}%. The cartilage explants were further analyzed to assess the penetration depth of the BPL molecules across the cartilage by using the same experimental setup. After 6 and 24 h, the cartilage explants were removed from the device, fixed, embedded in OCT (Cat. no. 27050; Ted Pella), sectioned into 15‐μm thick sections, and stained with DAPI to mark nuclei (Cat. no. P36971; Invitrogen). The sections were imaged with a Keyence BZ‐X710 fluorescence microscope. The penetration experiments were also repeated with bovine cartilage explants which were degraded with IL‐1β (Cat. no. 201‐LB‐005; R&D Systems).^39^ The IL‐1β was dissolved in chondrocyte media at a concentration of 25 ng/ml and new media containing IL‐1β was added every other day. After 4 days of incubation, the cartilage explants were placed in the PDMS molds, with G2 and G2HP BPL solutions at concentrations of 500 μg/ml. The uptake and penetration of the G2 and G2HP BPL solutions into the degraded cartilage was measured using the previously described Section 3.

#### 3.2.7 Desorption of the BPL molecules from the cartilage explants

The desorption experiments were carried out using cartilage explants following the adsorption of Cy5‐conjugated BPL (Cy5‐BPL) molecules. After 24 h of incubation with the Cy5‐BPL solution, the cartilage explants were washed with 1× PBS, moved into a 96‐well plate, and incubated in 200 μl of either 1× PBS or 10× PBS containing protease inhibitors (Cat. no. P8340; Sigma Aldrich). The protease inhibitors were used to prevent any explant degradation. The PBS solutions were collected following every 24 h for 4 days and replaced with fresh PBS. The fluorescent intensity of the collected PBS solutions was measured to determine the extent of BPL desorption over the 4 days of incubation time.

#### 3.2.8 Equilibrium uptake assay of the BPL molecules into cartilage explants

Cartilage explants (4 mm diameter × ~500 μm thickness) were incubated in 200 μl Cy5‐tagged BPL solution with varying initial concentrations (*C*_initial_) of 150, 300, 750, 1000, and 1500 μM (containing protease inhibitors) in a 96‐well plate at 37°C. The fluorescence intensity of the free solution, following 24 h, was quantified by the plate reader to obtain the final BPL concentration (*C*_final_). In addition, the cartilage explants were removed, washed with PBS, wiped, and weighed to obtain the wet weight. These cartilage explants were then lyophilized and weighed to obtain the dry weight.

The uptake ratio (*R*_*u*_) is defined as the concentration of the BPL molecules within the cartilage disc, which includes both bound (*C*_*B*_) and free (*C*_*F*_) BPL molecules, normalized by the concentration of BPL molecules in the free solution (*C*_final_). The equilibrium uptake assay was used to estimate the binding properties of the BPL molecules (i.e., equilibrium partition coefficient *K*, and equilibrium dissociation constant *K*_*D*_, and intra‐cartilage binding site concentration *N*) with the cartilage tissue following a first‐order, reversible, bimolecular interaction model as reported by Vedadghavami et al.^15^, ^34^ The *R*_*u*_ can be described as follows (Equation 1):

(1) $$R_{u}=\left(C_{B}+C_{F}\right)/C_{\text{final}}$$

The equilibrium dissociation constant, *K*_*D*_, can be related to *C*_*F*_, *C*_*B*_, and *N* to the following equation:

(2) $$K_{D}=\left\{\;C_{F}\times \left(N-C_{B}\right)\right\}/\mathrm{CB}$$

The equilibrium partition coefficient (*K*) of BPL is defined as:

(3) $$K=C_{F}/C_{\text{final}}$$

By combining the Equations ((1), (2), (3)), the value of *R*_*u*_ can be written as:

(4) $$R_{u}=K\left\{1+N/\left(K_{D}+{\mathrm{KC}}_{\text{final}}\right)\right\}$$

Fitting the Equation (4) with the experimentally obtained values of *R*_*u*_ and *C*_final_, the parameters *K*, *N*, and *K*_*D*_ were obtained.

#### 3.2.9 Cytotoxicity/viability assay after culturing bovine explants with BPL molecules

Bovine cartilage explants incubated in chondrocyte medium containing different concentrations of BPL molecules (11.5 and 23 μM). The explants were incubated for 24 h at 37°C and 5% CO_2_. After incubation, cartilage explants were manually sliced longitudinally into 100–200 μm thick sections, incubated with 2 μM calcein AM and 4 μM ethidium homodimer‐1 (Cat. no. L3224; Invitrogen) and imaged with a Keyence BZ‐X710 fluorescence microscope. Fluorescence images was quantified to determine the live (green) and dead (red) cells.^35^

#### 3.2.10 Biosynthesis of bovine cartilage explants cultured with G2HP BPL molecules

Bovine cartilage explants were cultured in chondrocyte medium containing G2HP BPL molecules at a concentration of 11.5 μM. At the initial time point, the explants were incubated with either chondrocyte medium or chondrocyte medium with G2HP BPL molecules at 37°C and 5% CO_2_. The medium was changed every other day. After 14 days of culture, cartilage explants (*n* = 3) from each group were fixed with 4% paraformaldehyde overnight. These explants were then dehydrated in 20% sucrose, embedded in OCT medium, and sectioned into 15 μm‐thick sections. After sectioning, the cartilage explants were stained with Safranin‐O, Fast Green, hematoxylin, and imaged with a Keyence BZ‐X710 fluorescence microscope.^37^ Cartilage explants at the initial time point were used as a control.

Additionally, biochemical analyses of the cartilage explants *via* 1,9‐dimethylmethylene blue (DMMB; Cat. no. 341088; MilliporeSigma) assay were used to quantify the proteoglycan content in the cartilage explants.^38^ Three cartilage explants were lyophilized, weighed for their dry weight, and digested in papain (Cat. no. LS003124; Worthington Biochem) overnight at 60°C. The papain digests of the cartilage were then diluted, mixed with DMMB dye and the absorbance of the solution at 525 nm was measured with a Tecan Infinite F200 Plate Reader. The chondroitin sulfate in each sample was quantified by comparison to a standard curve of chondroitin sulfate of varying concentrations and normalized to the dry weight of the cartilage explants.

#### 3.2.11 Localization of BPL molecules in ex vivo mouse knee joints

Following euthanasia, 12‐month‐old C57BL/6 male mouse knee joint explants were injected with saline, free Cy5 dye, or Cy5‐conjugated BPL molecules (G2HP BPL) in sterile saline (50 μl, 50 μg/ml; 1.9 μM). The concentration of the Cy5 in the free Cy5 group was calculated to be the same as the Cy5 content in the 50 μg/ml G2HP BPL solution. The knee joint explants were left in an incubator at 37°C and 5% CO_2_ for 24 h. After this, the explants were fixed, dehydrated in 30% sucrose, and embedded in OCT medium. Undecalcified joints were then cryosectioned using a Cryojane Tape‐Transfer system, counterstained with DAPI, and imaged to visualize the localization and distribution of the injected molecules.

#### 3.2.12 In vivo retention of BPL molecules in rat joint

All animal procedures were carried out in accordance with protocols approved by Duke University Institutional Animal Care and Use Committee (IACUC) in compliance with NIH guidelines for laboratory animal care (A151‐20‐07). Unilateral destabilization of the medial meniscus (DMM) surgery, which is often used to induce post‐traumatic osteoarthritis (PTOA), was performed in rats as previously described.^47^ Briefly, 12‐week‐old female Lewis rats (*n* = 4, Charles River) were anesthetized under isoflurane, shaved on both legs, and disinfected with three scrubbing routines of povidone iodine and 70% ethanol. Buprenorphine SR (1 mg/ml, ZooPharm) was injected subcutaneously at a dose of 0.5 mg/kg. The skin and joint capsule were incised, and the medial meniscotibial ligament was transected. Following transection, bupivacaine (0.5%, Hospira) was applied topically, and Vicryl 6‐0 sutures were used to close the skin incision. One week after surgery, cyanine 7 (Cy7)‐conjugated PEGylated BPL (G2HP BPL) molecules in sterile saline were administered (50 μl, 500 μg/ml; 19 μM) into injured and uninjured knees of the rat by intra‐articular injection. The fluorescent signal in the uninjured joint and in the DMM joint injury model were compared to examine the effect of injury‐mediated changes on the retention and penetration of the BPL molecules following intraarticular injection. An IVIS Kinetic System and Living Image software were used to serially acquire (excitation filter: 745 nm, emission filter: indocyanine green, ICG) and quantify fluorescence intensity within each joint over 28 days.^41^ Radiant efficiency data within a fixed anatomical region of interest (ROI) over time for each group were fit to a single‐phase exponential decay with a common plateau compared to untreated animal background fluorescence.

#### 3.2.13 Methylprednisolone release kinetics

The release kinetics of 6‐alpha‐methylprednisolone from the BPL molecules was carried out in PBS as a function of time over 14 days.^45^ The G2HP BPL molecules conjugated with the drug were dissolved in PBS at a concentration of 10 mg/ml. The solutions (10 mg/ml) were loaded into dialysis bags (MWCO = 2 kDa; Cat. no. D2272; Sigma Aldrich), which were then sealed and placed in a 15‐ml centrifuge tube with 5 ml of PBS. At predetermined time intervals (0, 2, 6, 12, 24, 48, 72, 96, 120, 144, 168, 240, 336, and 504 h), 1 ml of the solution from the outside of the dialysis bag was removed and supplemented with an equal amount of fresh PBS. The drug release from the BPL molecules was calculated by quantifying the methylprednisolone content in the collected solution *via* UV/Vis absorption spectroscopy (Genesys 10S UV–Vis Spectrophotometer and Semi‐Micro Cuvette, Cat. no. 9700‐590; VWR) as a function of time. A standard calibration curve of absorbance (at ~255 nm) as a function of methylprednisolone concentration (2.5–50 μg/ml in PBS) was used to calculate the concentration of the released methylprednisolone. Two different standard calibration curves of absorbance (at ~255 nm) as a function of the concentration of G2HP BPL molecules (2.5–50 μg/ml in PBS) and G2HP BPL molecules conjugated with methylprednisolone (15.625–500 μg/ml in PBS) were used to calculate methylprednisolone conjugation to the G2HP BPL molecules. To examine the effect of esterases on methylprednisolone release, we also used PBS containing 10% of FBS as an incubating medium. The G2HP conjugated with the drug was dissolved in PBS containing 10% FBS at a concentration of 10 mg/ml. The solution mixture was loaded into a dialysis bag with a MWCO of 2 kDa, sealed, and placed in a 15‐ml centrifuge tube with 5 ml of PBS containing 10% FBS. The release profile up to 3 days post‐incubation was determined using UV/Vis as described above (at ~260 nm).

### 3.3 Statistical analyses

All in vitro experiments were repeated at least twice and reproduced, where each individual experiment had a sample size of at least 3. All numerical data are expressed as means plus or minus standard error of the mean. Data were subjected to one‐way analysis of variance (ANOVA with post hoc Tukey–Kramer test for multiple comparisons), or two‐way analysis of variance (ANOVA with post hoc Bonferroni test for multiple comparisons). *p* values of <0.05 were considered statistically significant and indicated with an asterisk. For each figure, a table corresponding to their *p* values and corresponding significance are provided in the supplementary information. All statistical analyses were performed with GraphPad Prism 10.0.0.

## 4 CONCLUSION AND FUTURE DIRECTION

In this study, we describe the use of ring opening polymerization to synthesize different generations of branched poly(l‐lysine) (BPL) nanocarriers with similar hydrodynamic diameters while varying the number of poly(lysine) branches and net surface charge. The synthetic route offers a cost‐effective and scalable approach to generate cartilage‐penetrating nanocarriers which can be modified with drugs or other molecules. *In vitro* cartilage explant studies used both healthy and OA‐mimetic cartilage that was generated by using IL‐β cytokine challenge to examine the effect net charge of the nanocarrier on their transport across the cartilage tissue with different levels of proteoglycan content. PEGylation was used to screen the charge and improve the cytocompatibility as examined by chondrocyte viability. Increasing the net charge did not increase the uptake suggesting the existence of an optimal charge towards the penetration and retention of the nanocarriers within the cartilage tissue. Generation two BPL (G2HP BPL) molecules were further studied for their intra‐articular distribution and retention; longitudinal IVIS imaging showed that the BPL molecules were retained within the joint at least up to 14 days, although there were differences in half‐life between injured and uninjured joints. Conjugation and release studies involving methylprednisolone, a clinically used steroid, showed the potential to load therapeutic agents. PEGylation was shown to improve cell viability and prevent potential aggregation of the highly charged BPL molecules in synovial fluid. While the live/dead analyses of cartilage explants showed no cytotoxicity, it is important to ensure that the BPL molecules do not elicit any inflammatory response in the joint for their applications to joint diseases. Prior studies involving linear ε‐poly(lysine) and highly charged branched cationic molecules, though not lysine‐based, showed no synovitis and joint inflammation suggesting that these molecules could be compatible to delivery drugs to the joint.^12^, ^17^ Future studies include assessing the therapeutic efficacy of BPL molecules to deliver disease‐modifying agents to the cartilage tissue and validating its efficacy in pre‐clinical animal models of arthritis. These studies will also include detailed analyses of the compatibility of the BPL molecules following their intra‐articular administration.

## AUTHOR CONTRIBUTIONS

**Gavin Gonzales:** Conceptualization (supporting); data curation (equal); formal analysis (equal); investigation (equal); methodology (equal); writing – original draft (equal); writing – review and editing (equal). **Jiaul Hoque:** Conceptualization (equal); data curation (equal); formal analysis (equal); investigation (equal); methodology (equal); validation (equal); writing – original draft (equal); writing – review and editing (supporting). **Anna Gilpin:** Data curation (supporting); formal analysis (supporting); methodology (equal). **Biswanath Maity:** Data curation (supporting); investigation (supporting). **Stefan Zauscher:** Formal analysis (supporting); writing – review and editing (supporting). **Shyni Varghese:** Conceptualization (equal); formal analysis (supporting); funding acquisition (lead); investigation (supporting); project administration (lead); supervision (lead); writing – original draft (lead); writing – review and editing (lead).

## FUNDING INFORMATION

Shyni Varghese and Gavin Gonzales would like to thank funding from the National Institute of Arthritis and Musculoskeletal and Skin Diseases of the National Institutes of Health under Award Number NIH R01 AR079189 and R01 AR082809, and the National Institute on Aging of the National Institutes of Health under Award Number NIH R01 AG074491. Gavin Gonzales would like to acknowledge the Alfred P. Sloan Foundation (Sloan Scholar, Alfred P. Sloan Foundation's UCEM Program, 2019); Gavin Gonzales and Anna Gilpin would like to acknowledge the support by the National Science Foundation Graduate Research Fellowship Program under Grant No. DGE 1644868.

## CONFLICT OF INTEREST STATEMENT

The authors have no conflicts of interest to declare.

### PEER REVIEW

The peer review history for this article is available at https://www.webofscience.com/api/gateway/wos/peer-review/10.1002/btm2.10612.

## Supporting information


**DATA S1.** Supporting Information

## Data Availability

The data that support the findings of this study are available from the corresponding author upon reasonable request.
