# Supplementary material for: Branched poly‐l‐lysine for cartilage penetrating carriers
Source: Bioeng Transl Med. 2023 Oct 16;9(3):e10612. doi: 10.1002/btm2.10612 (PMC11135149; doi:10.1002/btm2.10612)
Supplement: Supplementary file 1 — DATA S1. Supporting Information [file BTM2-9-e10612-s001.docx]

Supporting Information for

**Branched poly-L-lysine for cartilage penetrating carriers**

Gavin Gonzales^1#^, Jiaul Hoque^2#^, Anna Gilpin^1^, Biwsanath Maity^2^, Stefan Zauscher ^3^ and Shyni Varghese^1, 2, 3^

^1^Department of Biomedical Engineering, Duke University, Durham, NC 27710, USA

^2^Department of Orthopaedic Surgery, Duke University School of Medicine, Durham, NC 27710, USA

^3^Department of Mechanical Engineering and Materials Science, Duke University, Durham, NC 27710, USA

#Contributed equally

*To whom correspondence should be addressed:

E-mail: [shyni.varghese@duke.edu](mailto:shyni.varghese@duke.edu), Tel: +1-919-660-5273


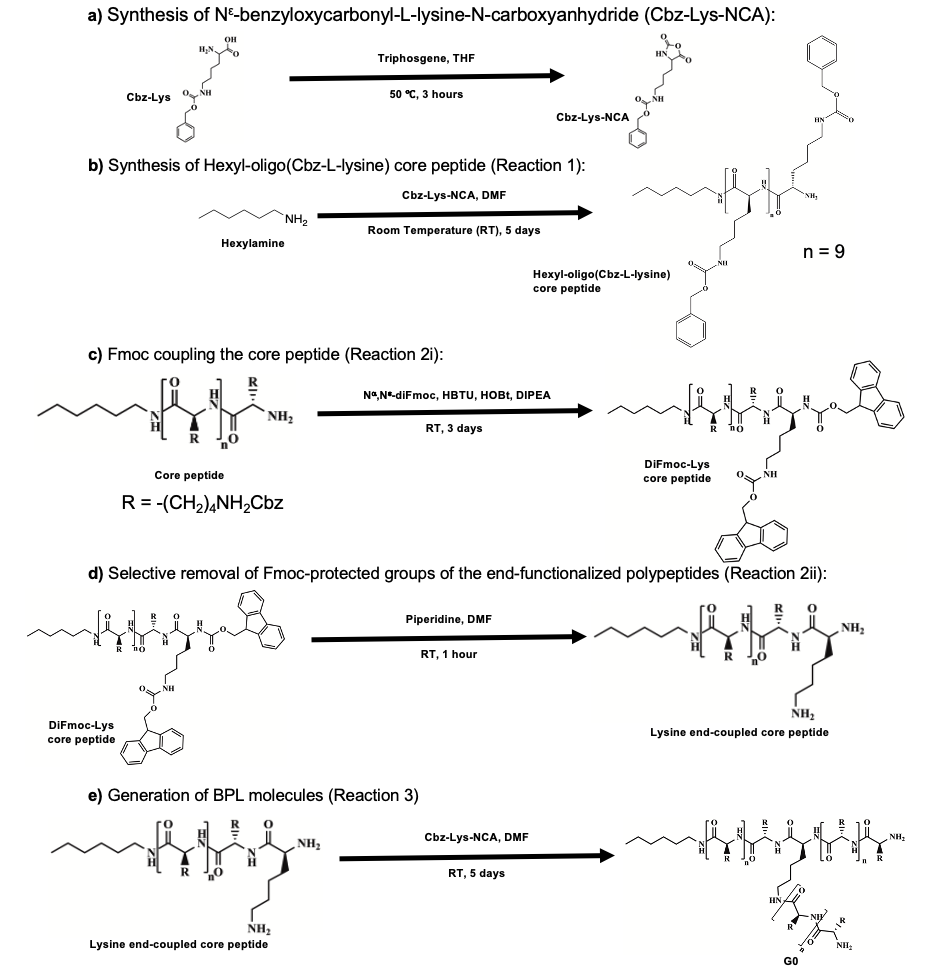


**Figure S1**. (a) Synthesis of Lys-NCA-Cbz monomer. (b) Synthesis of the core peptide using hexylamine as an initiator. The core peptides contain 10 lysine repeating units. (c) N^α^,N^ε^-di(9-fluorenylmethoxycarbonyl-L-lysine (N^α^,N^ε^-diFmoc-Lys) coupling to the core peptide. (d) Selective removal of the diFmoc groups to obtain lysine end-coupled core peptide. (e) Synthesis of the G0 peptide by using lysine end-coupled core peptide with Cbz-Lys-NCA *via* ring-opening polymerization.


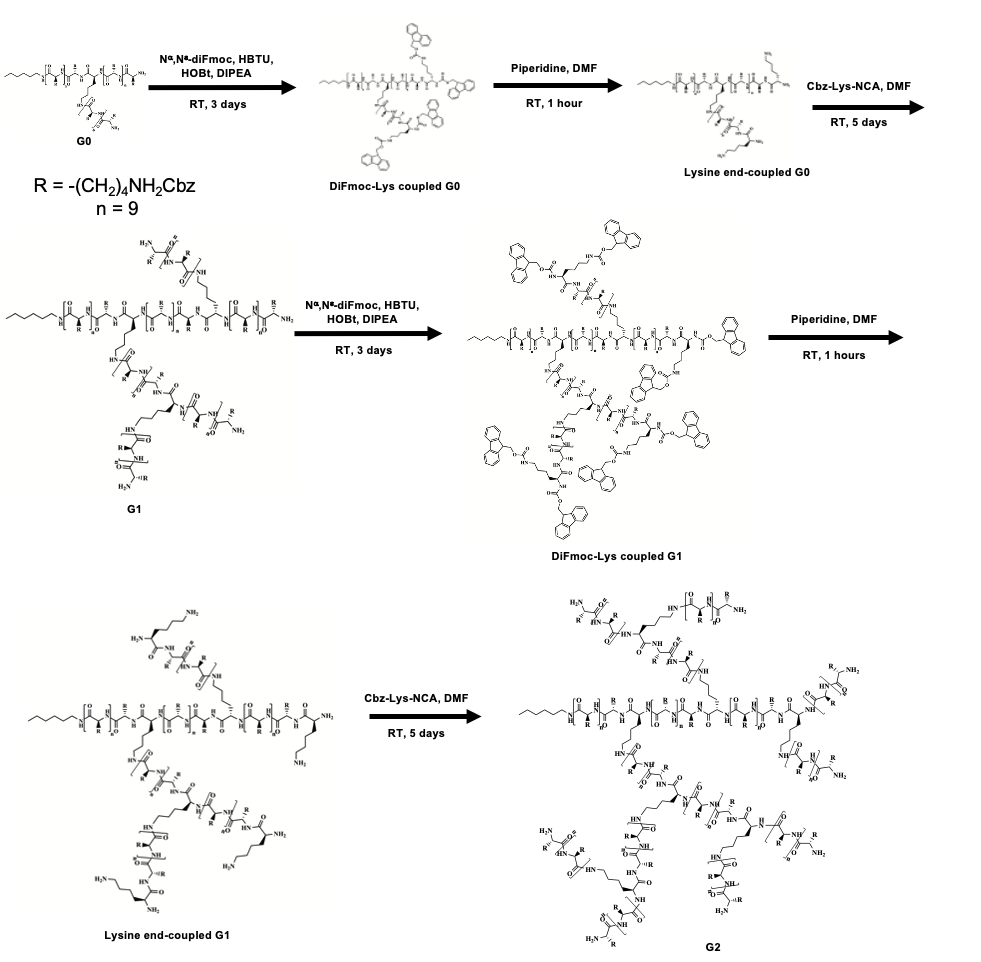


**Figure S2**. Synthesis of higher generation of BPL molecules. Reaction scheme showing the synthesis of G1 and G2 molecules from G0.


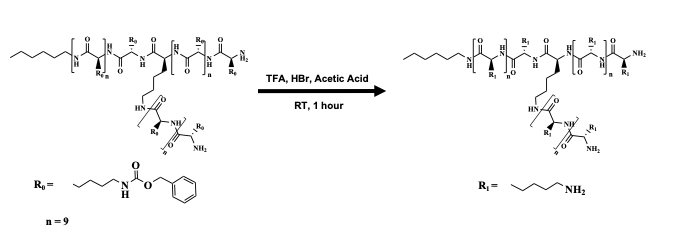


**Figure S3.** Deprotection of BPL molecules to remove Cbz protecting groups.

**Table S1.** Theoretical characterization of branched poly(L-lysine) molecules assuming each arm contains 10 lysine repeating units.

| Poly(L-lysine) | Number of arms | Theoretical number of lysine units per molecule |
| --- | --- | --- |
| Core | 1 | 10 |
| G0 | 2 | 30 |
| G1 | 4 | 70 |
| G2 | 8 | 150 |


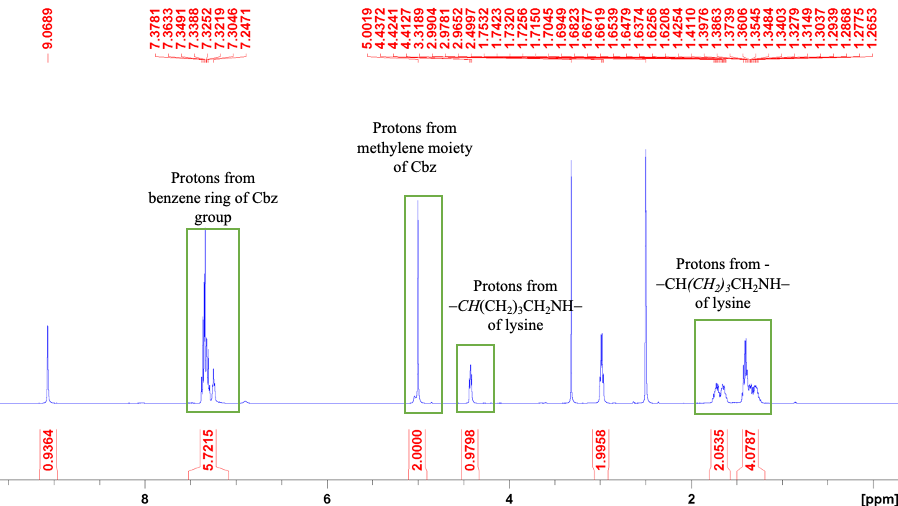


**Figure S4**. ^1^HNMR spectrum of Cbz-Lys-NCA in DMSO-d_6_ at 25 °C. The green boxes indicate the peaks corresponding to the protons of Cbz-protected lysine.


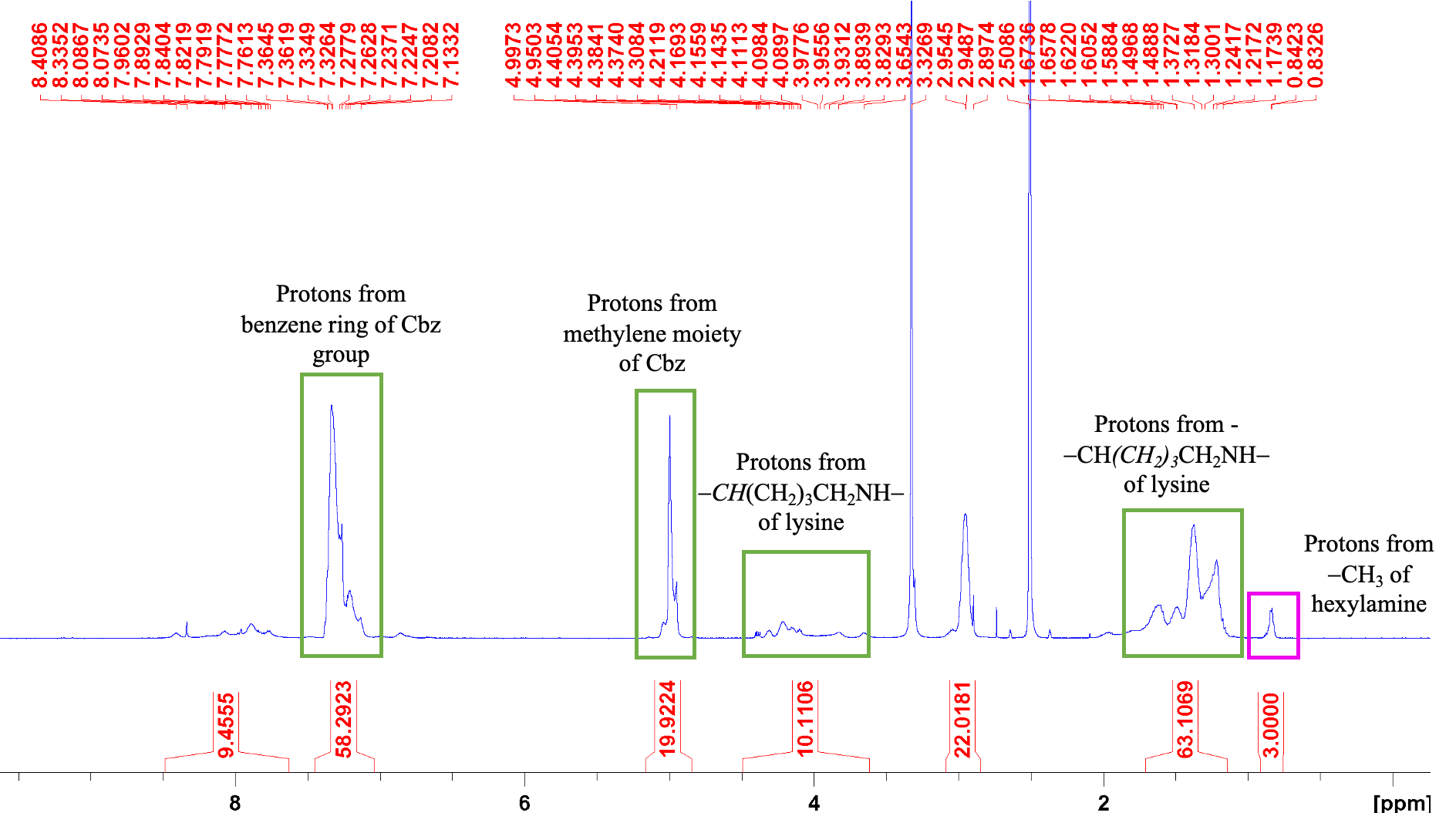


**Figure S5**. ^1^HNMR spectrum of Cbz-protected core peptide in DMSO-d_6_ at 25 °C. The pink box indicates the peak corresponding to the terminal −CH_3_ protons of the hexylamine initiator. The green boxes indicate representative peaks corresponding to the protons of Cbz-protected lysine units.


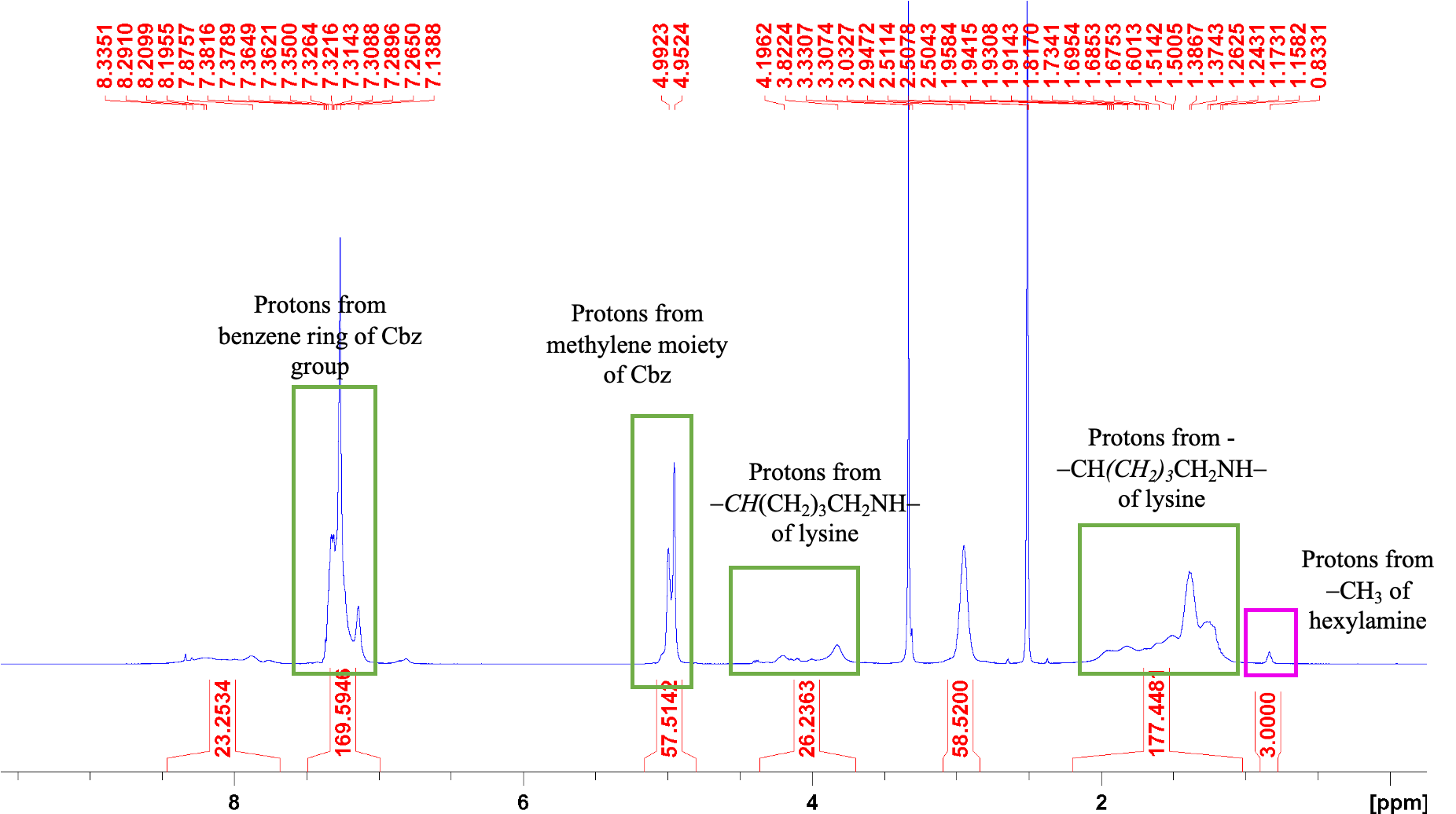


**Figure S6**. ^1^HNMR spectrum of Cbz-protected G0 in DMSO-d_6_ at 25 °C. The pink box indicates the peak corresponding to the terminal −CH_3_ protons of the hexylamine initiator. The green boxes indicate representative peaks corresponding to the Cbz-protected protected lysine units.


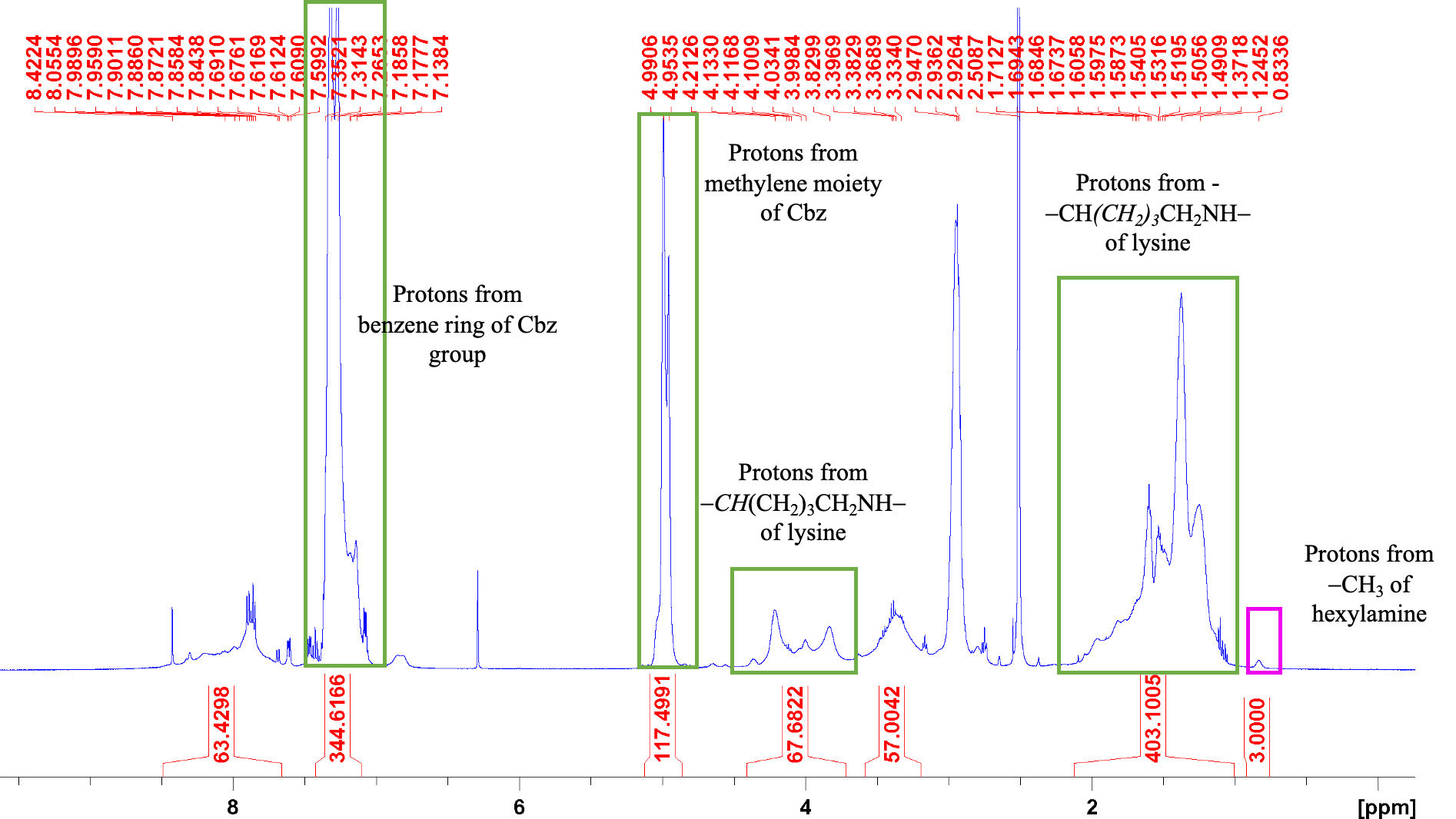


**Figure S7**. ^1^HNMR spectrum of Cbz-protected G1 in DMSO-d_6_ at 25 °C. The pink box indicates the peak corresponding to the terminal −CH_3_ protons of the hexylamine initiator. The green boxes indicate the peaks corresponding to the protons of Cbz-protected lysine units.


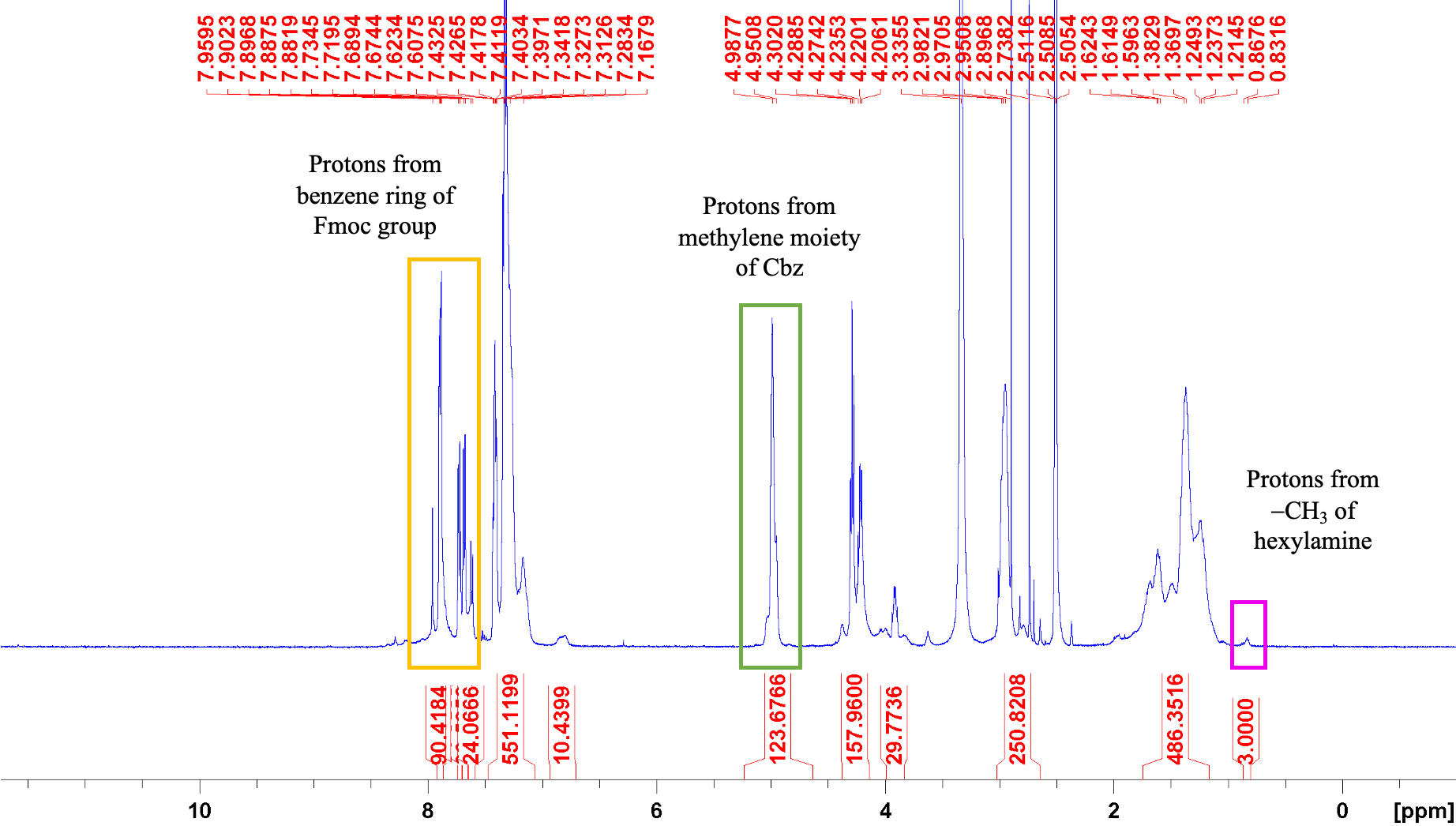


**Figure S8**. ^1^HNMR spectrum of Cbz-protected, N^α^,N^ε^-diFmoc-Lys conjugated G1 in DMSO-d_6_ at 25 °C. The pink box indicates the peak corresponding to the terminal −CH_3_ protons of the hexylamine initiator. The yellow box indicates the peaks corresponding to the protons of the benzene ring of N^α^,N^ε^-diFmoc-Lys units. The green box indicates the peak corresponding to the protons of Cbz-protected lysine units.


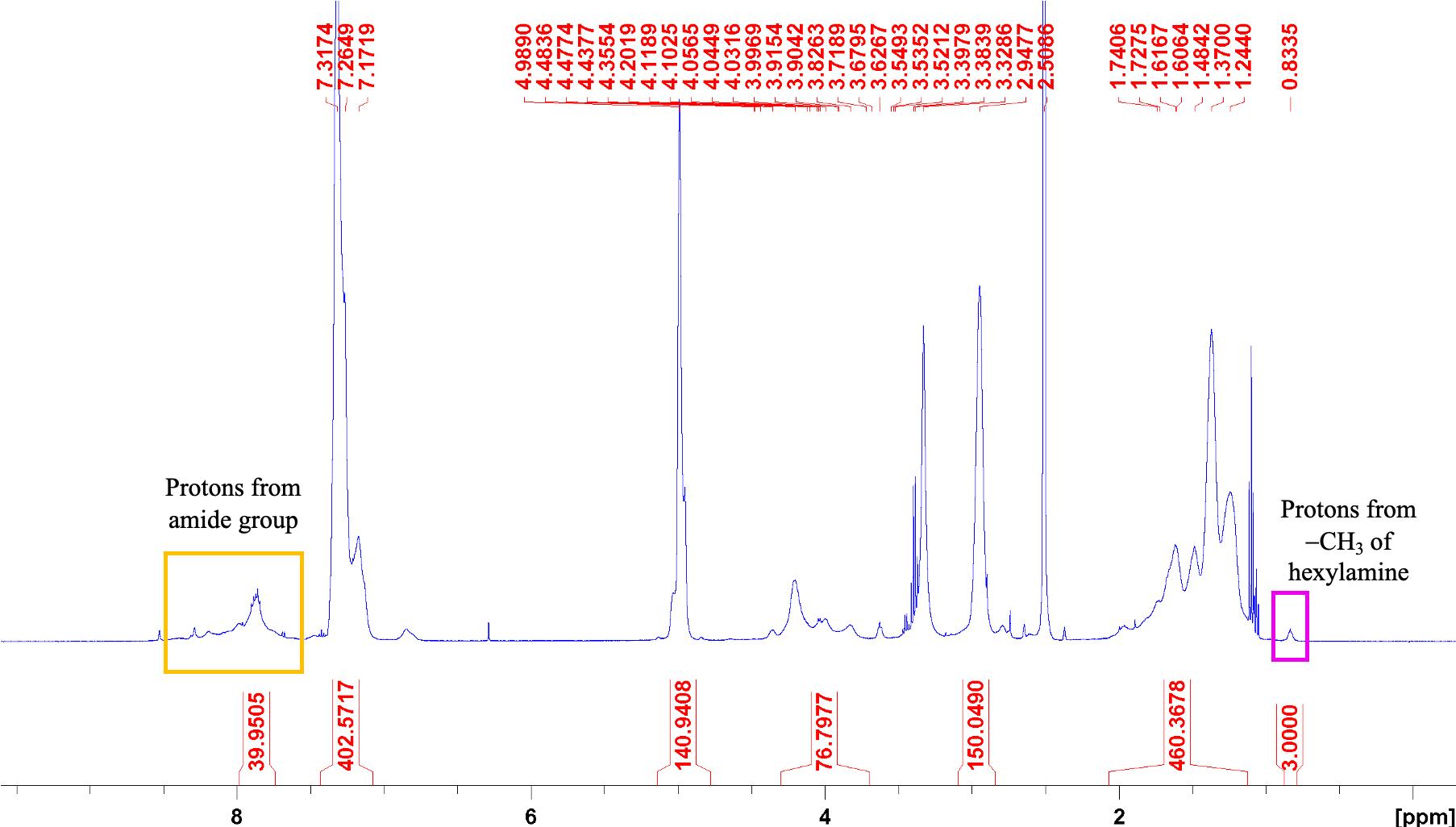


**Figure S9**. ^1^HNMR spectrum of Cbz-protected G1 following selective removal of the diFmoc groups in DMSO-d_6_ at 25 °C. The pink box indicates the peak corresponding to the terminal −CH_3_ protons of the hexylamine initiator. The peaks in the yellow box indicate the protons of the −CONH− group.


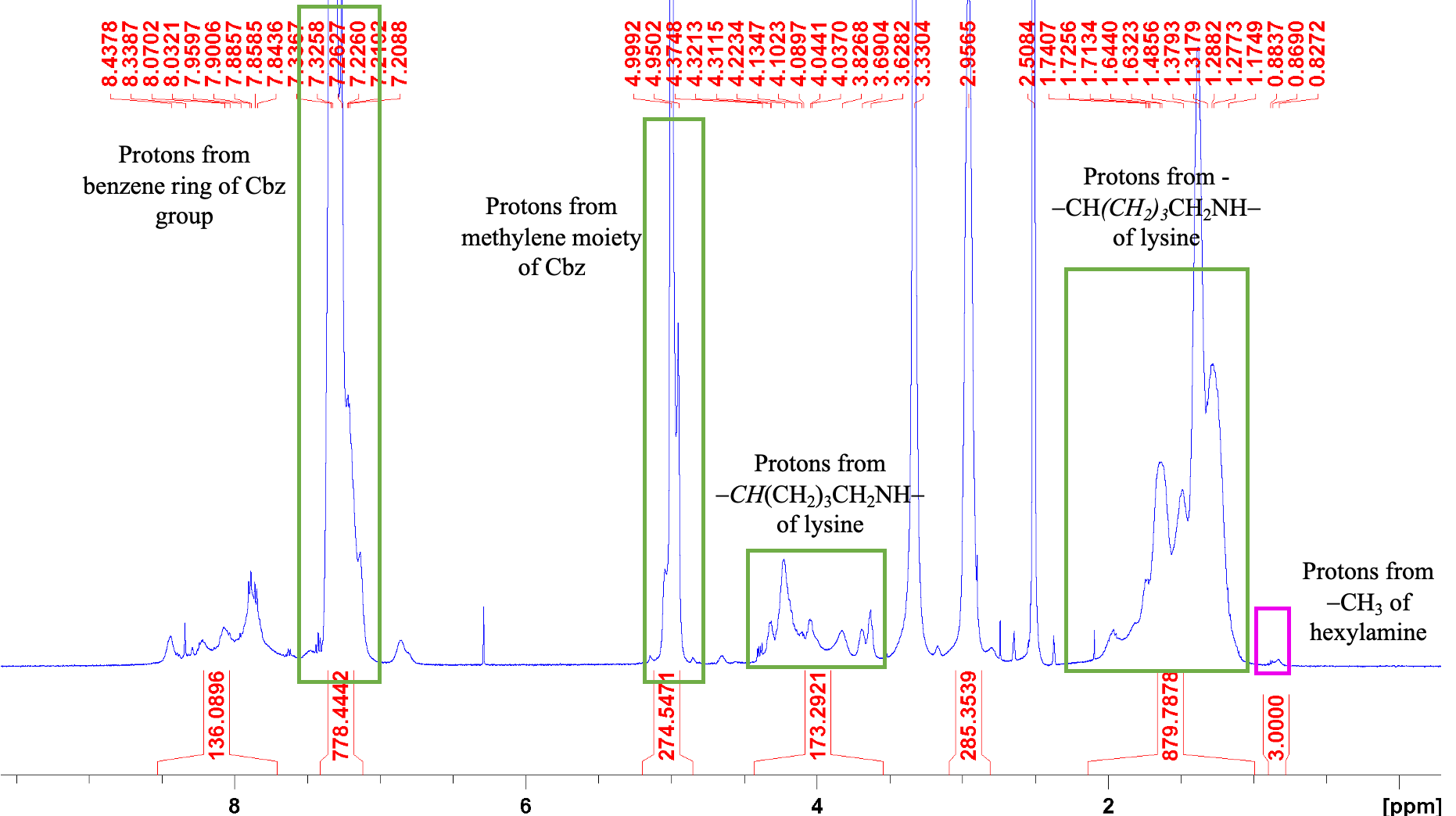


**Figure S10**. ^1^HNMR spectrum of Cbz-protected G2 recorded in DMSO-d_6_ at 25 °C. The pink box indicates the peak corresponding to the terminal −CH_3_ protons of the hexylamine initiator. The green boxes indicate the peaks corresponding to the protons of Cbz-protected lysine units.


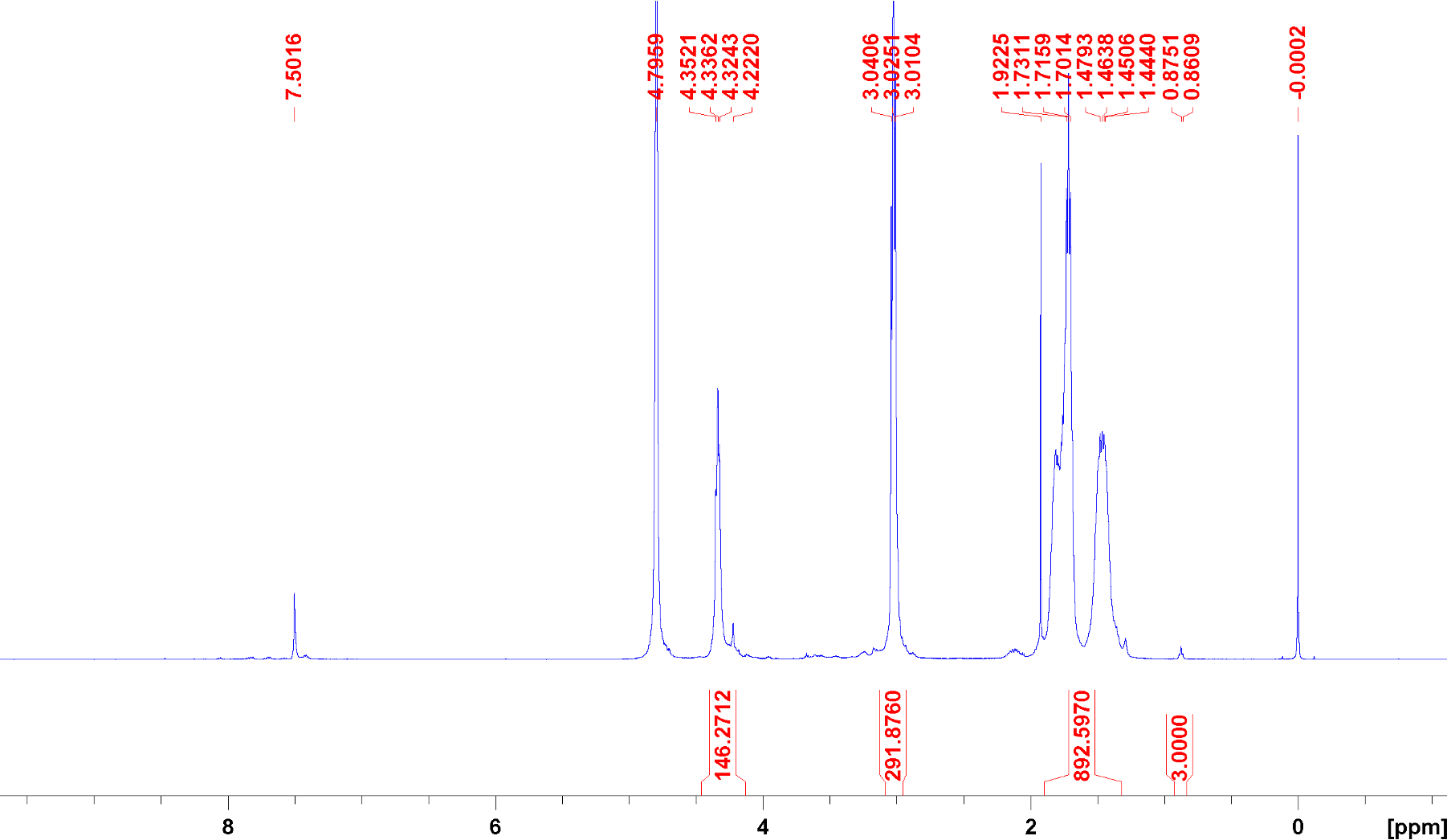


**Figure S11**. ^1^HNMR spectrum of fully deprotected G2 in D_2_O at 25 °C.


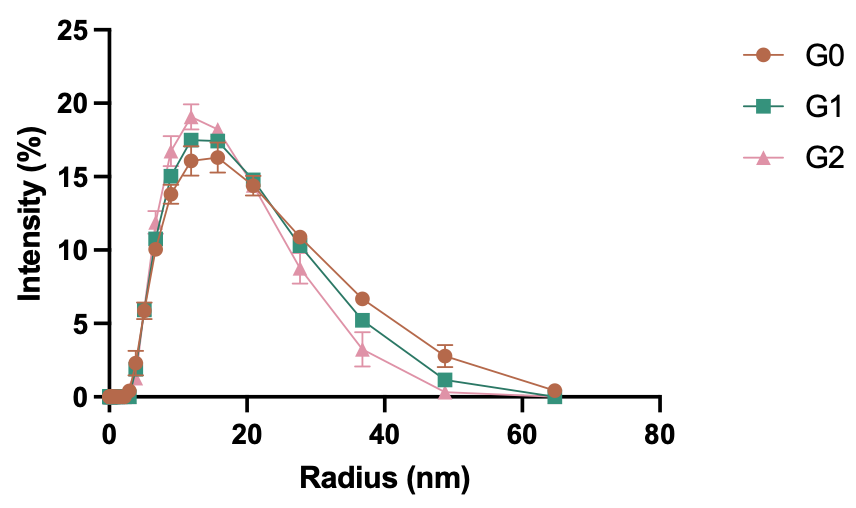


**Figure S12.** Histogram of DLS measurements of G0, G1 and G2 BPL molecules.


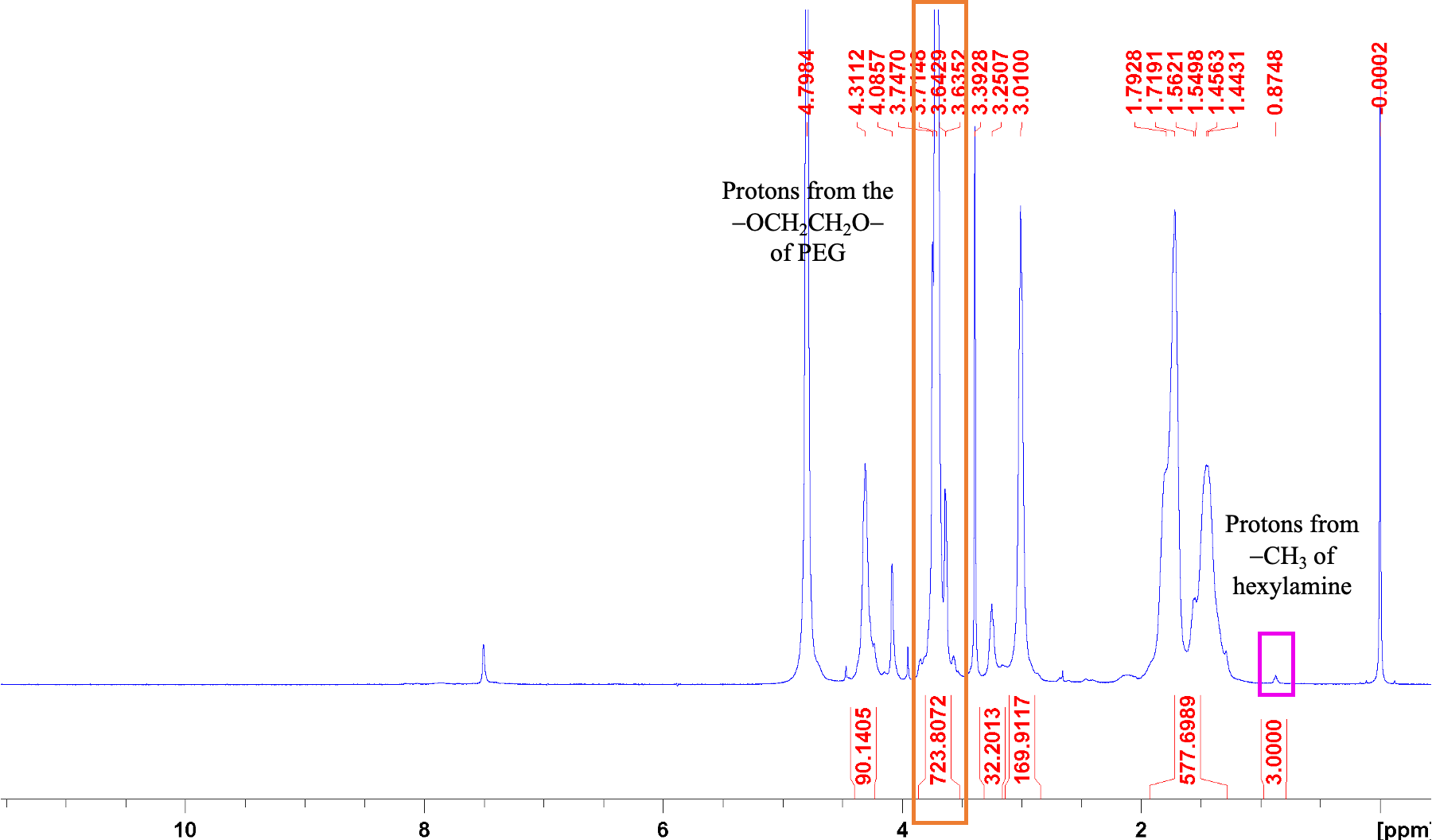


**Figure S13**. ^1^HNMR spectrum of G1LP in D_2_O at 25 °C. The pink box indicates the peak corresponding to the terminal −CH_3_ protons of the hexylamine initiator. The orange box indicates the peaks corresponding to the protons of −OCH_2_CH_2_O− unit of PEG chains.


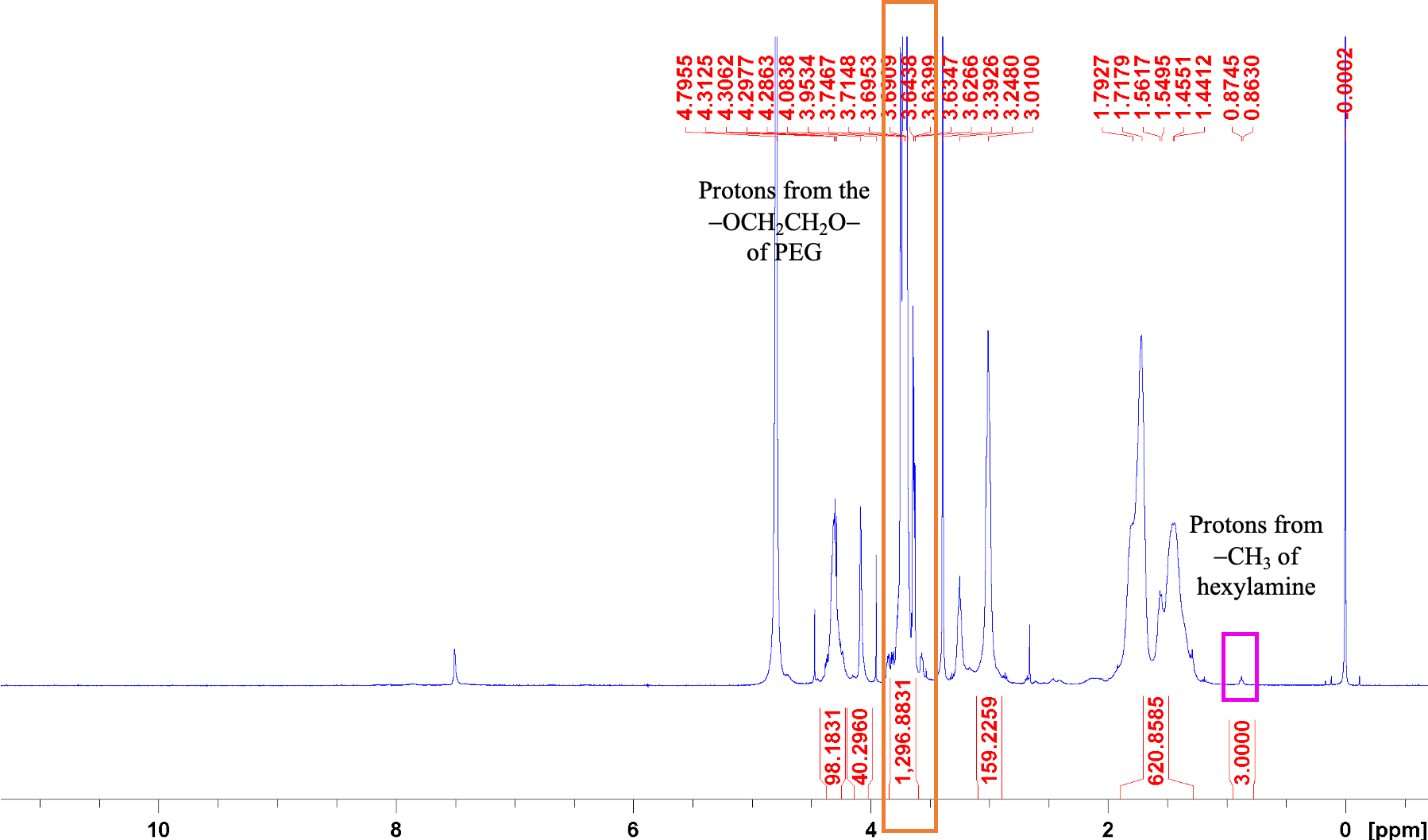


**Figure S14**. ^1^HNMR spectrum of G1HP recorded in D_2_O at 25 °C. The pink box indicates the peak corresponding to the terminal −CH_3_ protons of the hexylamine initiator. The orange box indicates the peaks corresponding to the protons of −OCH_2_CH_2_O− unit of PEG chains.


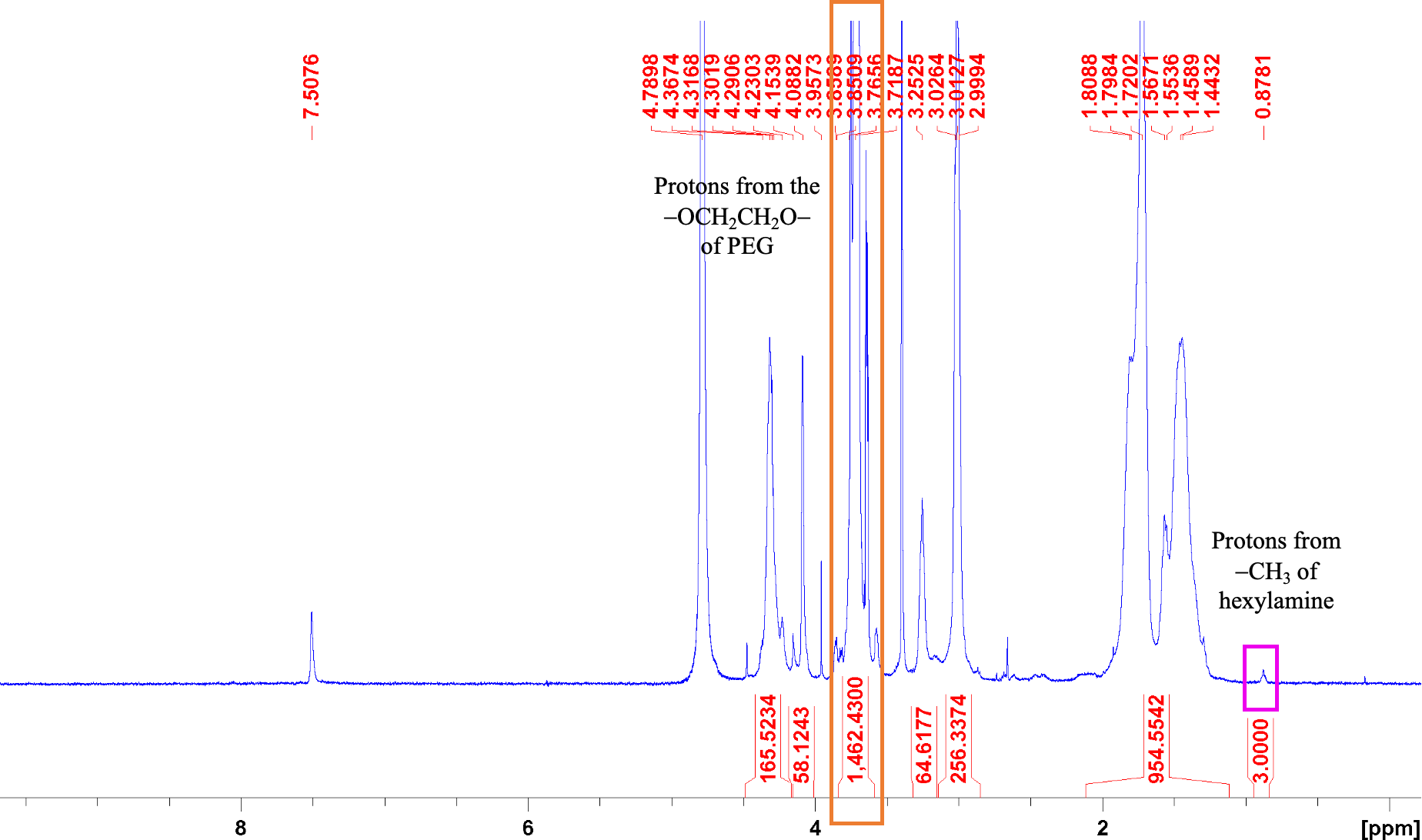


**Figure S15**. ^1^HNMR spectrum of G2LP in D_2_O at 25 °C. The pink box indicates the peak corresponding to the terminal −CH_3_ protons of hexylamine initiator. The orange box indicates the peaks corresponding to the protons of −OCH_2_CH_2_O− unit of PEG chains.


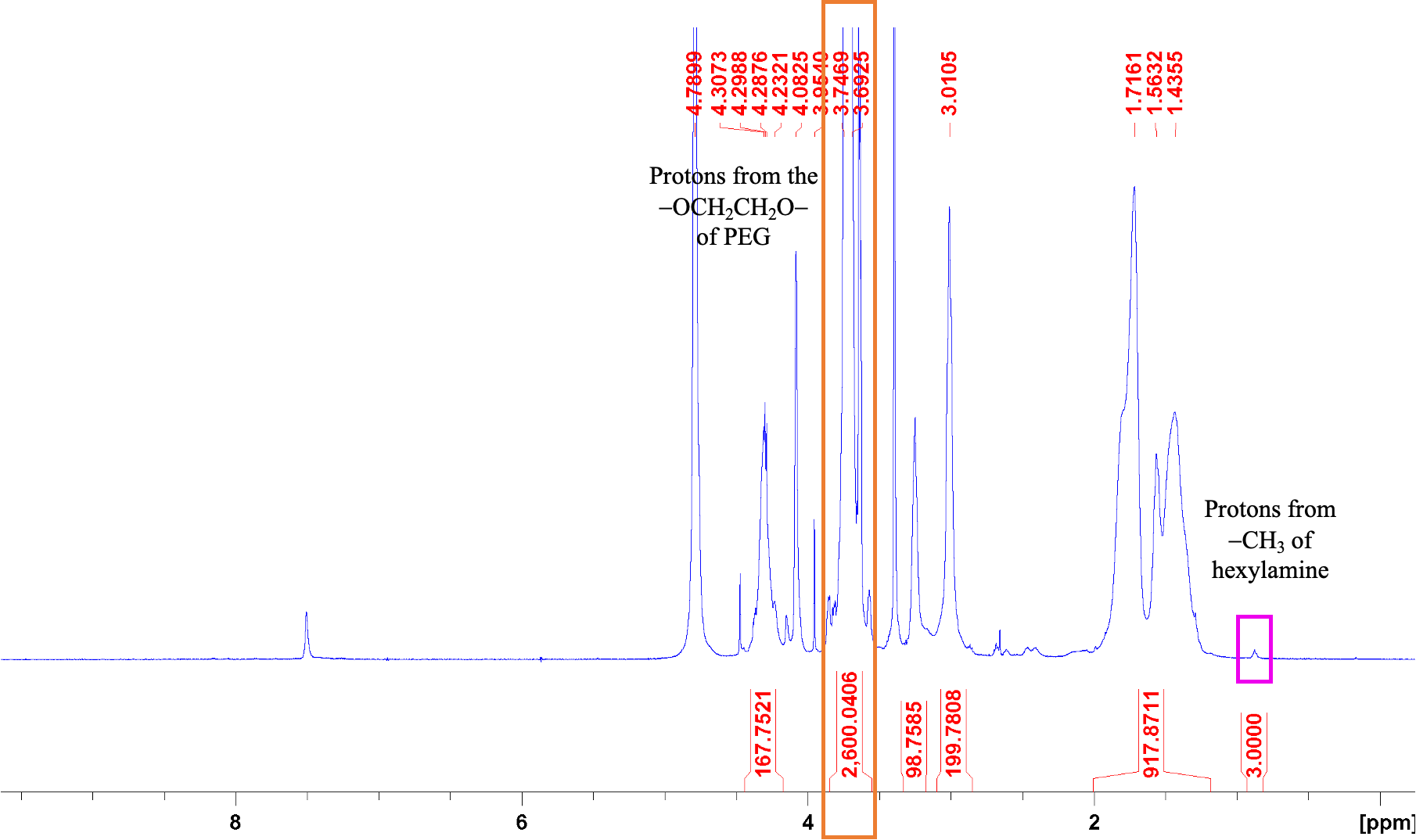


**Figure S16**. ^1^HNMR spectrum of G2HP in D_2_O at 25 °C. The pink box indicates the peak corresponding to the terminal −CH_3_ protons of the hexylamine initiator. The orange box indicates the peaks corresponding to the protons of −OCH_2_CH_2_O− unit of PEG chains.

**Table S2.** Multiple comparisons from one-way ANOVA with Tukey post-hoc test from uptake of BPL molecules to cartilage after 24 hours (Figure 2b).

| Tukey's multiple comparisons test | Mean Diff. | 95.00% CI of diff. | Summary | Adjusted P Value |
| --- | --- | --- | --- | --- |
| G0 vs. G1 | 3.490 | -9.594 to 16.57 | ns | 0.9647 |
| G0 vs. G1LP | 3.756 | -9.328 to 16.84 | ns | 0.9504 |
| G0 vs. G1HP | 10.02 | -3.065 to 23.10 | ns | 0.1933 |
| G0 vs. G2 | -5.553 | -18.64 to 7.531 | ns | 0.7680 |
| G0 vs. G2LP | 10.26 | -2.821 to 23.35 | ns | 0.1748 |
| G0 vs. G2HP | 10.17 | -2.911 to 23.26 | ns | 0.1815 |
| G1 vs. G1LP | 0.2664 | -12.82 to 13.35 | ns | >0.9999 |
| G1 vs. G1HP | 6.529 | -6.555 to 19.61 | ns | 0.6245 |
| G1 vs. G2 | -9.043 | -22.13 to 4.041 | ns | 0.2832 |
| G1 vs. G2LP | 6.774 | -6.311 to 19.86 | ns | 0.5872 |
| G1 vs. G2HP | 6.683 | -6.401 to 19.77 | ns | 0.6010 |
| G1LP vs. G1HP | 6.263 | -6.822 to 19.35 | ns | 0.6649 |
| G1LP vs. G2 | -9.310 | -22.39 to 3.775 | ns | 0.2560 |
| G1LP vs. G2LP | 6.507 | -6.577 to 19.59 | ns | 0.6278 |
| G1LP vs. G2HP | 6.417 | -6.668 to 19.50 | ns | 0.6416 |
| G1HP vs. G2 | -15.57 | -28.66 to -2.488 | * | 0.0155 |
| G1HP vs. G2LP | 0.2444 | -12.84 to 13.33 | ns | >0.9999 |
| G1HP vs. G2HP | 0.1541 | -12.93 to 13.24 | ns | >0.9999 |
| G2 vs. G2LP | 15.82 | 2.732 to 28.90 | * | 0.0138 |
| G2 vs. G2HP | 15.73 | 2.642 to 28.81 | * | 0.0144 |
| G2LP vs. G2HP | -0.09025 | -13.17 to 12.99 | ns | >0.9999 |

**Table S3.** Multiple comparisons from one-way ANOVA with Tukey post-hoc test from penetration of BPL molecules to cartilage after 6 hours (Figure 3b).

| Tukey's multiple comparisons test | Mean Diff. | 95.00% CI of diff. | Summary | Adjusted P Value |
| --- | --- | --- | --- | --- |
| G0 vs. G1 | 93.33 | 20.84 to 165.8 | ** | 0.0084 |
| G0 vs. G1LP | 75.00 | 2.502 to 147.5 | * | 0.0405 |
| G0 vs. G1HP | -21.67 | -94.16 to 50.83 | ns | 0.9406 |
| G0 vs. G2 | 68.33 | -4.165 to 140.8 | ns | 0.0707 |
| G0 vs. G2LP | 35.00 | -37.50 to 107.5 | ns | 0.6568 |
| G0 vs. G2HP | -23.33 | -95.83 to 49.16 | ns | 0.9183 |
| G1 vs. G1LP | -18.33 | -90.83 to 54.16 | ns | 0.9726 |
| G1 vs. G1HP | -115.0 | -187.5 to -42.50 | ** | 0.0014 |
| G1 vs. G2 | -25.00 | -97.50 to 47.50 | ns | 0.8915 |
| G1 vs. G2LP | -58.33 | -130.8 to 14.16 | ns | 0.1565 |
| G1 vs. G2HP | -116.7 | -189.2 to -44.17 | ** | 0.0012 |
| G1LP vs. G1HP | -96.67 | -169.2 to -24.17 | ** | 0.0063 |
| G1LP vs. G2 | -6.667 | -79.16 to 65.83 | ns | 0.9999 |
| G1LP vs. G2LP | -40.00 | -112.5 to 32.50 | ns | 0.5199 |
| G1LP vs. G2HP | -98.33 | -170.8 to -25.84 | ** | 0.0055 |
| G1HP vs. G2 | 90.00 | 17.50 to 162.5 | * | 0.0112 |
| G1HP vs. G2LP | 56.67 | -15.83 to 129.2 | ns | 0.1775 |
| G1HP vs. G2HP | -1.667 | -74.16 to 70.83 | ns | >0.9999 |
| G2 vs. G2LP | -33.33 | -105.8 to 39.16 | ns | 0.7018 |
| G2 vs. G2HP | -91.67 | -164.2 to -19.17 | ** | 0.0097 |
| G2LP vs. G2HP | -58.33 | -130.8 to 14.16 | ns | 0.1565 |

**Table S4.** Multiple comparisons from one-way ANOVA with Tukey post-hoc test from penetration of BPL molecules to cartilage after 24 hours (Figure 3c).

| Tukey's multiple comparisons test | Mean Diff. | 95.00% CI of diff. | Summary | Adjusted P Value |
| --- | --- | --- | --- | --- |
| G0 vs. G1 | -176.7 | -275.6 to -77.72 | *** | 0.0004 |
| G0 vs. G1LP | -188.3 | -287.3 to -89.39 | *** | 0.0002 |
| G0 vs. G1HP | -173.3 | -272.3 to -74.39 | *** | 0.0005 |
| G0 vs. G2 | -71.67 | -170.6 to 27.28 | ns | 0.2400 |
| G0 vs. G2LP | -81.67 | -180.6 to 17.28 | ns | 0.1395 |
| G0 vs. G2HP | -50.00 | -148.9 to 48.95 | ns | 0.6118 |
| G1 vs. G1LP | -11.67 | -110.6 to 87.28 | ns | 0.9995 |
| G1 vs. G1HP | 3.333 | -95.61 to 102.3 | ns | >0.9999 |
| G1 vs. G2 | 105.0 | 6.055 to 203.9 | * | 0.0344 |
| G1 vs. G2LP | 95.00 | -3.945 to 193.9 | ns | 0.0636 |
| G1 vs. G2HP | 126.7 | 27.72 to 225.6 | ** | 0.0088 |
| G1LP vs. G1HP | 15.00 | -83.95 to 113.9 | ns | 0.9981 |
| G1LP vs. G2 | 116.7 | 17.72 to 215.6 | * | 0.0166 |
| G1LP vs. G2LP | 106.7 | 7.721 to 205.6 | * | 0.0310 |
| G1LP vs. G2HP | 138.3 | 39.39 to 237.3 | ** | 0.0043 |
| G1HP vs. G2 | 101.7 | 2.721 to 200.6 | * | 0.0423 |
| G1HP vs. G2LP | 91.67 | -7.279 to 190.6 | ns | 0.0778 |
| G1HP vs. G2HP | 123.3 | 24.39 to 222.3 | * | 0.0109 |
| G2 vs. G2LP | -10.00 | -108.9 to 88.95 | ns | 0.9998 |
| G2 vs. G2HP | 21.67 | -77.28 to 120.6 | ns | 0.9865 |
| G2LP vs. G2HP | 31.67 | -67.28 to 130.6 | ns | 0.9202 |

**Table S5.** Multiple comparisons from one-way ANOVA with Tukey post-hoc test from desorption of BPL molecules from cartilage after 4 days in 1x PBS (Figure 4a).

| Tukey's multiple comparisons test | Mean Diff. | 95.00% CI of diff. | Summary | Adjusted P Value |
| --- | --- | --- | --- | --- |
| G0 vs. G1 | 8.220 | 1.001 to 15.44 | * | 0.0213 |
| G0 vs. G1LP | 8.594 | 1.376 to 15.81 | * | 0.0154 |
| G0 vs. G1HP | 7.656 | 0.4376 to 14.87 | * | 0.0345 |
| G0 vs. G2 | 10.89 | 3.675 to 18.11 | ** | 0.0022 |
| G0 vs. G2LP | 5.879 | -1.339 to 13.10 | ns | 0.1482 |
| G0 vs. G2HP | 4.770 | -2.448 to 11.99 | ns | 0.3275 |
| G1 vs. G1LP | 0.3747 | -6.844 to 7.593 | ns | >0.9999 |
| G1 vs. G1HP | -0.5637 | -7.782 to 6.655 | ns | >0.9999 |
| G1 vs. G2 | 2.673 | -4.545 to 9.892 | ns | 0.8568 |
| G1 vs. G2LP | -2.341 | -9.559 to 4.878 | ns | 0.9157 |
| G1 vs. G2HP | -3.450 | -10.67 to 3.769 | ns | 0.6664 |
| G1LP vs. G1HP | -0.9384 | -8.157 to 6.280 | ns | 0.9992 |
| G1LP vs. G2 | 2.299 | -4.920 to 9.517 | ns | 0.9219 |
| G1LP vs. G2LP | -2.715 | -9.934 to 4.503 | ns | 0.8481 |
| G1LP vs. G2HP | -3.824 | -11.04 to 3.394 | ns | 0.5630 |
| G1HP vs. G2 | 3.237 | -3.981 to 10.46 | ns | 0.7236 |
| G1HP vs. G2LP | -1.777 | -8.995 to 5.442 | ns | 0.9759 |
| G1HP vs. G2HP | -2.886 | -10.10 to 4.332 | ns | 0.8106 |
| G2 vs. G2LP | -5.014 | -12.23 to 2.205 | ns | 0.2785 |
| G2 vs. G2HP | -6.123 | -13.34 to 1.095 | ns | 0.1226 |
| G2LP vs. G2HP | -1.109 | -8.328 to 6.109 | ns | 0.9980 |

**Table S6.** Multiple comparisons from one-way ANOVA with Tukey post-hoc test from desorption of BPL molecules from cartilage after 4 days in 10x PBS (Figure 4d).

| Tukey's multiple comparisons test | Mean Diff. | 95.00% CI of diff. | Summary | Adjusted P Value |
| --- | --- | --- | --- | --- |
| G0 vs. G1 | -8.963 | -38.95 to 21.02 | ns | 0.9406 |
| G0 vs. G1LP | -6.648 | -36.63 to 23.34 | ns | 0.9857 |
| G0 vs. G1HP | -9.905 | -39.89 to 20.08 | ns | 0.9089 |
| G0 vs. G2 | -0.3237 | -30.31 to 29.66 | ns | >0.9999 |
| G0 vs. G2LP | -7.215 | -37.20 to 22.77 | ns | 0.9784 |
| G0 vs. G2HP | -8.822 | -38.80 to 21.16 | ns | 0.9446 |
| G1 vs. G1LP | 2.315 | -27.67 to 32.30 | ns | >0.9999 |
| G1 vs. G1HP | -0.9420 | -30.93 to 29.04 | ns | >0.9999 |
| G1 vs. G2 | 8.640 | -21.34 to 38.62 | ns | 0.9496 |
| G1 vs. G2LP | 1.748 | -28.24 to 31.73 | ns | >0.9999 |
| G1 vs. G2HP | 0.1419 | -29.84 to 30.13 | ns | >0.9999 |
| G1LP vs. G1HP | -3.257 | -33.24 to 26.73 | ns | 0.9997 |
| G1LP vs. G2 | 6.325 | -23.66 to 36.31 | ns | 0.9889 |
| G1LP vs. G2LP | -0.5671 | -30.55 to 29.42 | ns | >0.9999 |
| G1LP vs. G2HP | -2.173 | -32.16 to 27.81 | ns | >0.9999 |
| G1HP vs. G2 | 9.582 | -20.40 to 39.56 | ns | 0.9207 |
| G1HP vs. G2LP | 2.690 | -27.29 to 32.67 | ns | >0.9999 |
| G1HP vs. G2HP | 1.084 | -28.90 to 31.07 | ns | >0.9999 |
| G2 vs. G2LP | -6.892 | -36.87 to 23.09 | ns | 0.9828 |
| G2 vs. G2HP | -8.498 | -38.48 to 21.49 | ns | 0.9532 |
| G2LP vs. G2HP | -1.606 | -31.59 to 28.38 | ns | >0.9999 |


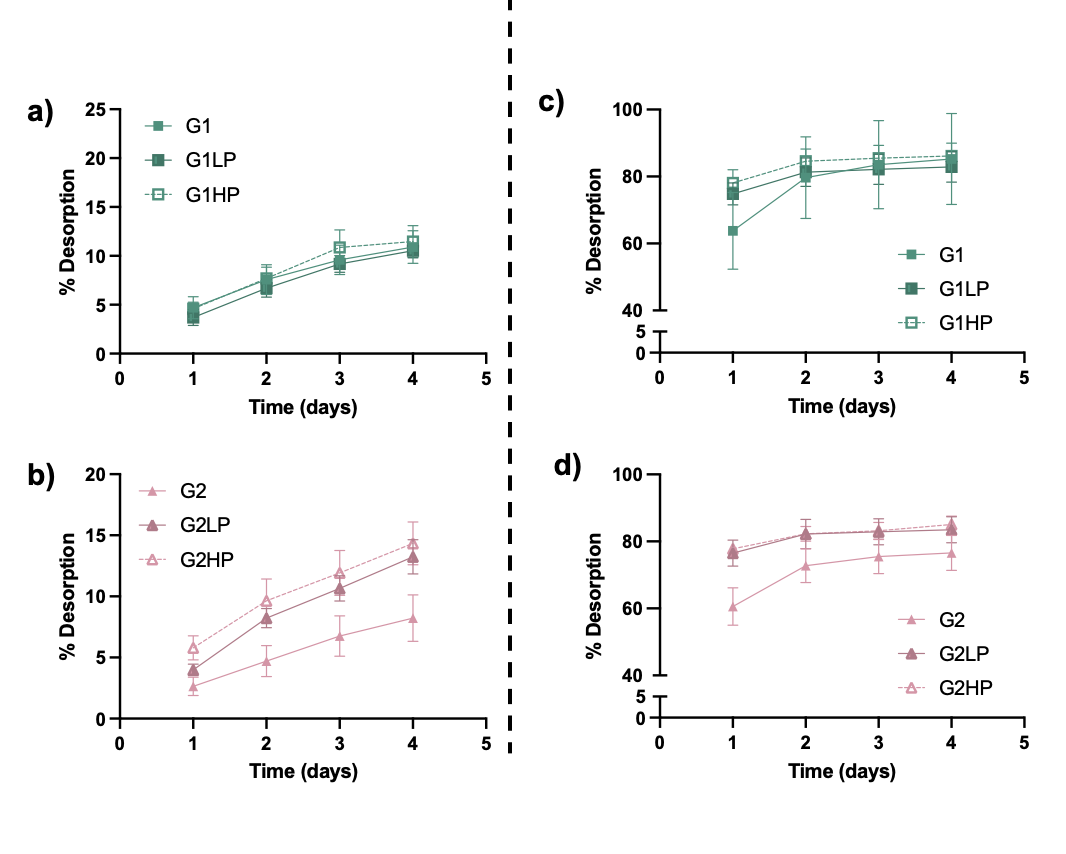


**Figure S17.** Desorption of BPL molecules from the cartilage explant. (a, b) Comparison of desorption of the BPL molecules based on PEGylation in 1X PBS. (c, d) Comparison of desorption of the BPL molecules based on PEGylation in 10X PBS.

**
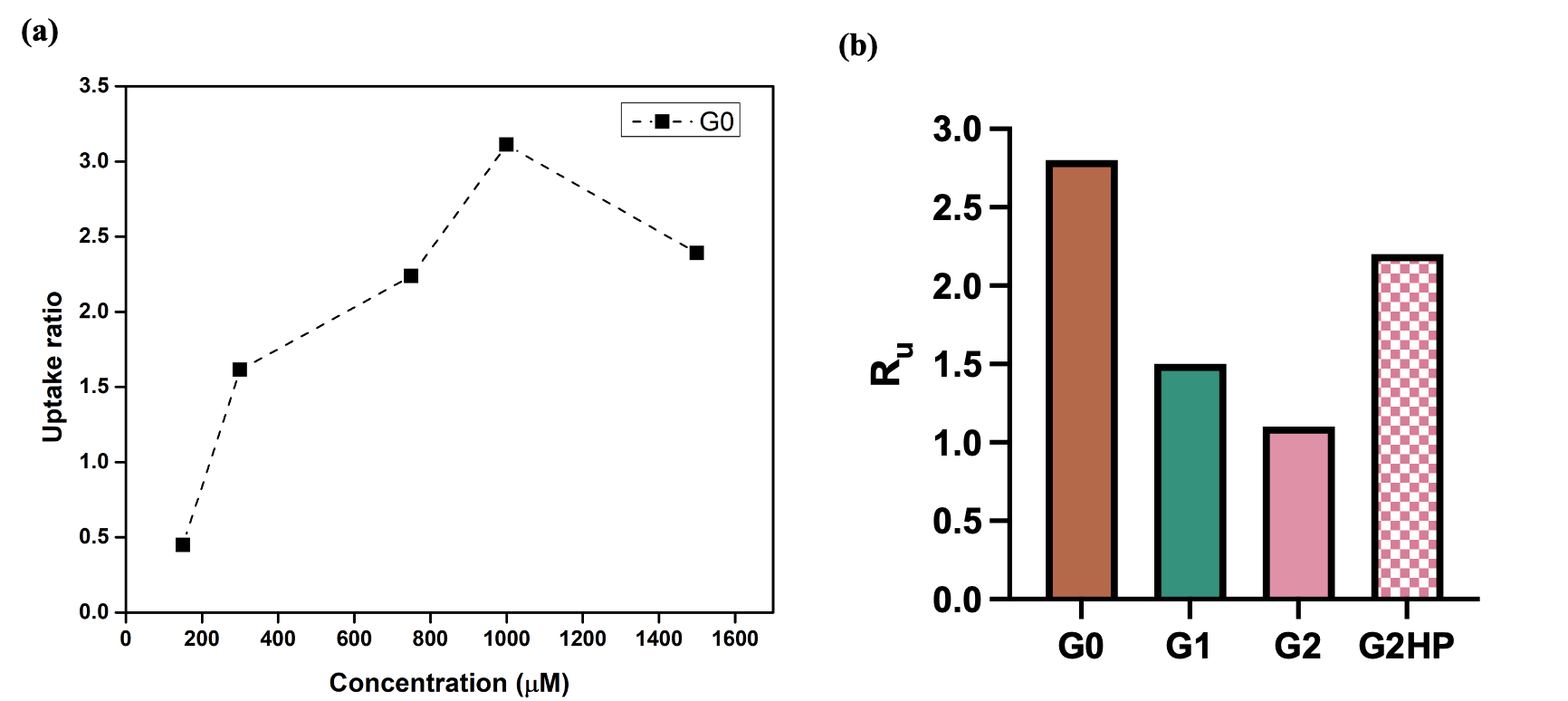
**

**Figure S18.** Intra-cartilage equilibrium uptake of BPL molecules. (a) Representative figure showing the cartilage uptake ratio of G0 BPL at varying concentrations. (b) Equilibrium uptake ratio (R_u_) for all the BPL molecules.

**Table S7.** Estimated equilibrium partition coefficient K, intra-cartilage equilibrium dissociation constant K_D_, and binding site concentration N from the equilibrium uptake assay.

| Poly(L-lysine) | Equilibrium partition coefficient (K) | Equilibrium dissociation constant (K_D_) (µM) | | Intra-cartilage binding site concentration (N) (µM) | |
| --- | --- | --- | --- | --- | --- |
| G0 | ~2.8 | | ~221 | | ~708 |
| G1 | ~1.5 | | ~1301 | | ~284 |
| G2 | ~1.1 | | ~6574 | | ~77 |
| G2HP | ~2.2 | | ~247 | | ~384 |


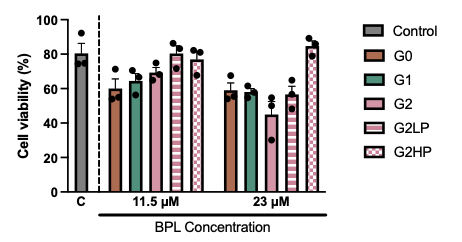


**Figure S19.** Percentage of viable cells in cartilage explants after 24 hours of incubation with BPL molecules in chondrocyte media. C: Control (i.e., Cartilage explants cultured in chondrocyte medium without the presence of BPL molecules).

**Table S8.** Multiple comparisons from one-way ANOVA with Tukey post-hoc test from live-dead viability/cytotoxicity assay after 24 hours of incubation of cartilage explants with BPL molecules at a concentration of 11.5 µM (Figure 5b).

| Tukey's multiple comparisons test | Mean Diff. | 95.00% CI of diff. | Summary | Adjusted P Value |
| --- | --- | --- | --- | --- |
| Control vs. G2 | 10.59 | -6.907 to 28.09 | ns | 0.3143 |
| Control vs. G2LP | -0.4533 | -17.95 to 17.05 | ns | 0.9998 |
| Control vs. G2HP | 2.923 | -14.58 to 20.42 | ns | 0.9567 |
| G2 vs. G2LP | -11.05 | -31.25 to 9.161 | ns | 0.3949 |
| G2 vs. G2HP | -7.670 | -27.88 to 12.54 | ns | 0.6726 |
| G2LP vs. G2HP | 3.377 | -16.83 to 23.58 | ns | 0.9567 |

**Table S9.** Multiple comparisons from one-way ANOVA with Tukey post-hoc test from proteoglycan biosynthesis assay, after incubation of BPL molecules and cartilage explants for 14 days (Figure 5d).

| Tukey's multiple comparisons test | Mean Diff. | 95.00% CI of diff. | Summary | Adjusted P Value |
| --- | --- | --- | --- | --- |
| Day 0 vs. Day 14 | 18.06 | -82.99 to 119.1 | ns | 0.8511 |
| Day 0 vs. Day 14+G2HP | 14.26 | -86.80 to 115.3 | ns | 0.9034 |
| Day 14 vs. Day 14+G2HP | -3.803 | -104.9 to 97.25 | ns | 0.9927 |

**
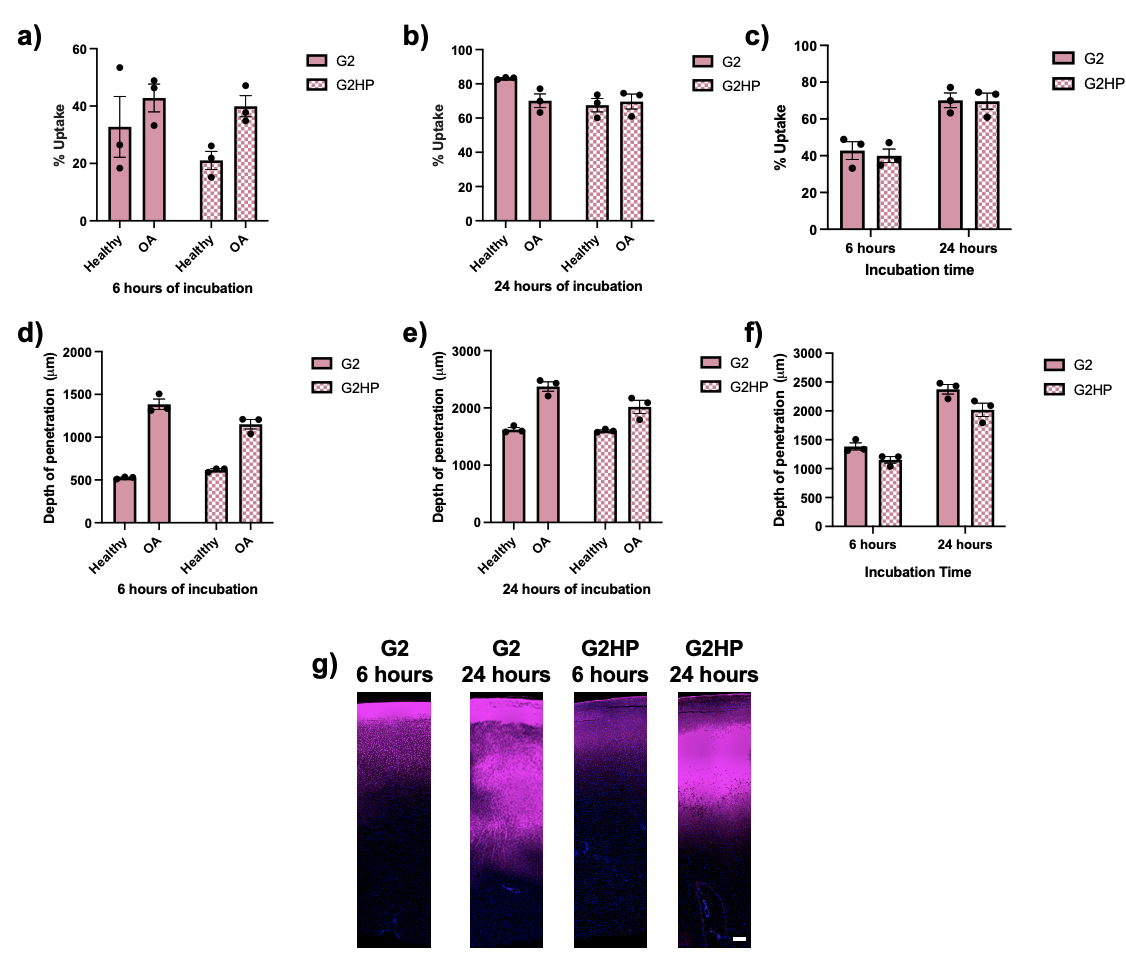
**

**Figure S20.** BPL uptake by the healthy and cytokine challenged cartilage explants. (a and b) BPL uptake by the healthy and OA-mimetic cartilage explants at 6 and 24 hours post-incubation. (c) Total BPL uptake by the OA-mimetic cartilage explants at 6 and 24 hours post-incubation. (d and e) Penetration depth of BPL molecules within the healthy and OA-mimetic cartilage explants after 6 and 24 hours. (f) Penetration depth of BPL molecules within the OA-mimetic cartilage explants after 6 and 24 hours. (g) Fluorescent images of G2 and G2HP BPL molecules within the OA-mimetic cartilage explants (Blue: DAPI, Purple: Cy5; Scale bar: 200 µm). Statistical analyses for (a), (b), (c), (d), (e), and (f) are provided in Table S10, S11, S12, S13, S14, and S15, respectively.

**Table S10.** Multiple comparisons from two-way ANOVA with Bonferroni's post-hoc test from BPL uptake into healthy and cytokine challenged cartilage explants after 6 hours (Figure S20a).

| Bonferroni's multiple comparisons test | Mean Diff. | 95.00% CI of diff. | Summary | Adjusted P Value |
| --- | --- | --- | --- | --- |
|  |  |  |  |  |
| Healthy - OA |  |  |  |  |
| G2 | -10.05 | -34.61 to 14.51 | ns | 0.5856 |
| G2HP | -18.87 | -43.43 to 5.683 | ns | 0.1347 |

**Table S11.** Multiple comparisons from two-way ANOVA with Bonferroni's post-hoc test from BPL uptake into healthy and cytokine challenged cartilage explants after 24 hours (Figure S20b).

| Bonferroni's multiple comparisons test | Mean Diff. | 95.00% CI of diff. | Summary | Adjusted P Value |
| --- | --- | --- | --- | --- |
|  |  |  |  |  |
| Healthy - OA |  |  |  |  |
| G2 | 13.12 | -0.7245 to 26.97 | ns | 0.0625 |
| G2HP | -2.102 | -15.95 to 11.74 | ns | >0.9999 |

**Table S12.** Multiple comparisons from two-way ANOVA with Bonferroni’s post-hoc test from BPL uptake into cytokine challenged cartilage explants (Figure S20c).

| Bonferroni's multiple comparisons test | Mean Diff. | 95.00% CI of diff. | Summary | Adjusted P Value |
| --- | --- | --- | --- | --- |
|  |  |  |  |  |
| G2 - G2HP |  |  |  |  |
| 6 hours | 2.877 | -13.63 to 19.39 | ns | >0.9999 |
| 24 hours | 0.5033 | -16.01 to 17.01 | ns | >0.9999 |

**Table S13.** Multiple comparisons from two-way ANOVA with Bonferroni’s post-hoc test from BPL penetration into healthy and cytokine challenged cartilage explants ay 6 hours (Figure S20d).

| Bonferroni's multiple comparisons test | Mean Diff. | 95.00% CI of diff. | Summary | Adjusted P Value |
| --- | --- | --- | --- | --- |
|  |  |  |  |  |
| Healthy - OA |  |  |  |  |
| G2 | -859.7 | -1023 to -696.0 | **** | <0.0001 |
| G2HP | -534.8 | -698.5 to -371.1 | **** | <0.0001 |

**Table S14.** Multiple comparisons from two-way ANOVA with Bonferroni’s post-hoc test from BPL penetration into healthy and cytokine challenged cartilage explants at 24 hours (Figure S20e).

| Bonferroni's multiple comparisons test | Mean Diff. | 95.00% CI of diff. | Summary | Adjusted P Value |
| --- | --- | --- | --- | --- |
|  |  |  |  |  |
| Healthy - OA |  |  |  |  |
| G2 | -751.6 | -1038 to -465.6 | *** | 0.0002 |
| G2HP | -419.4 | -705.4 to -133.3 | ** | 0.0075 |

**Table S15.** Multiple comparisons from two-way ANOVA with Bonferroni’s post-hoc test from BPL penetration into cytokine challenged cartilage explants at 6 and 24 hours (Figure S20f).

| Bonferroni's multiple comparisons test | Mean Diff. | 95.00% CI of diff. | Summary | Adjusted P Value |
| --- | --- | --- | --- | --- |
|  |  |  |  |  |
| G2 - G2HP |  |  |  |  |
| 6 hours | 233.2 | -86.58 to 552.9 | ns | 0.1594 |
| 24 hours | 353.9 | 34.12 to 673.6 | * | 0.0319 |


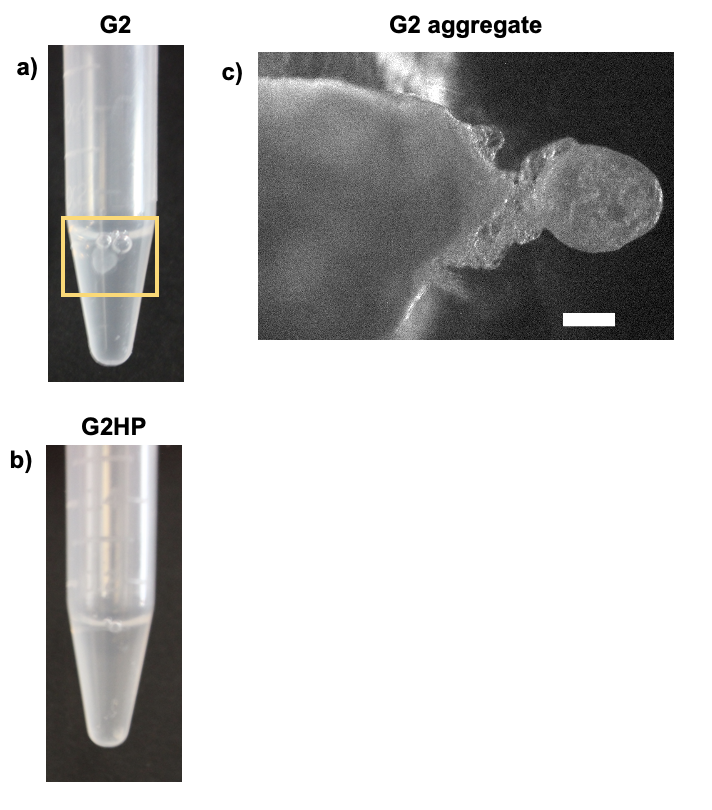


**Figure S21.** (a, c) Non-PEGylated G2-BPL molecules showed aggregation in 1X PBS supplemented with synovial fluid and (b) PEGylation limited the aggregation of the nanocarriers. Scale bar: 50 µm.

**Table S16.** Multiple comparisons from two-way ANOVA with Bonferroni’s post-hoc test to compare the uninjured and DMM groups from longitudinal IVIS study over 28 days (Figure 6d).

| Bonferroni's multiple comparisons test | Mean Diff. | 95.00% CI of diff. | Summary | Adjusted P Value |
| --- | --- | --- | --- | --- |
|  |  |  |  |  |
| Uninjured - DMM |  |  |  |  |
| 0 | 0.000 | -20.66 to 20.66 | ns | >0.9999 |
| 1 | 0.8378 | -19.83 to 21.50 | ns | >0.9999 |
| 2 | 15.76 | -4.905 to 36.42 | ns | 0.2727 |
| 4 | 27.76 | 7.095 to 48.42 | ** | 0.0029 |
| 6 | 37.96 | 17.30 to 58.63 | **** | <0.0001 |
| 14 | 16.25 | -4.412 to 36.92 | ns | 0.2328 |
| 23 | 5.970 | -14.69 to 26.63 | ns | >0.9999 |
| 30 | 4.714 | -15.95 to 25.38 | ns | >0.9999 |


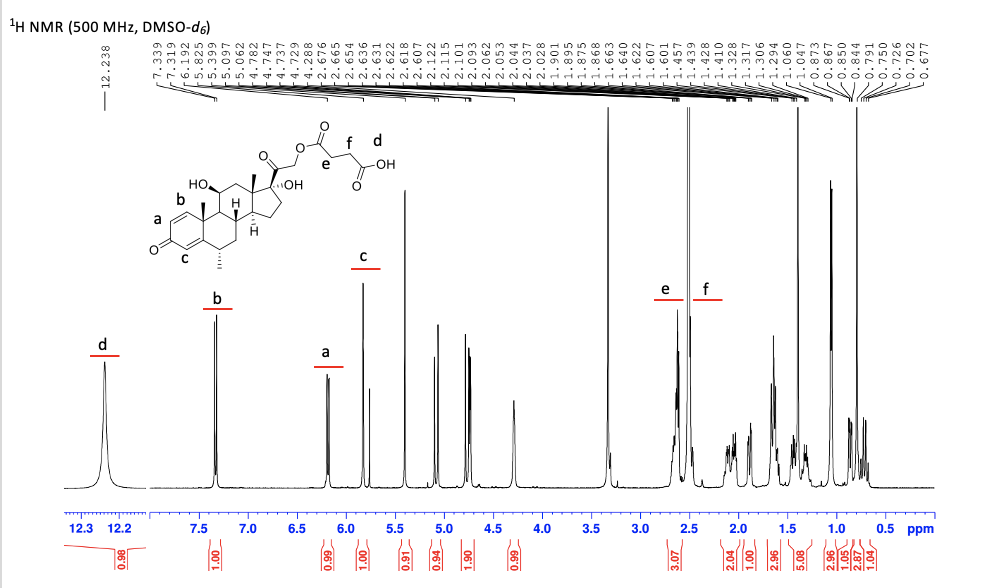


**Figure S22.** ^1^HNMR spectrum of succinic acid conjugated methylprednisolone in DMSO-d_6_.


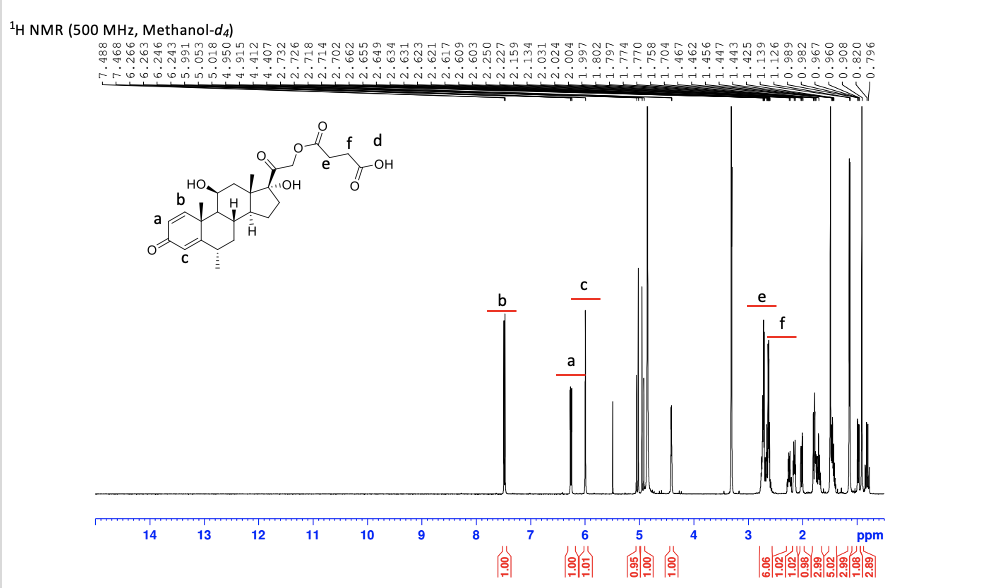


**Figure S23.** ^1^HNMR spectrum of succinic acid conjugated methylprednisolone in methanol-d_4_.


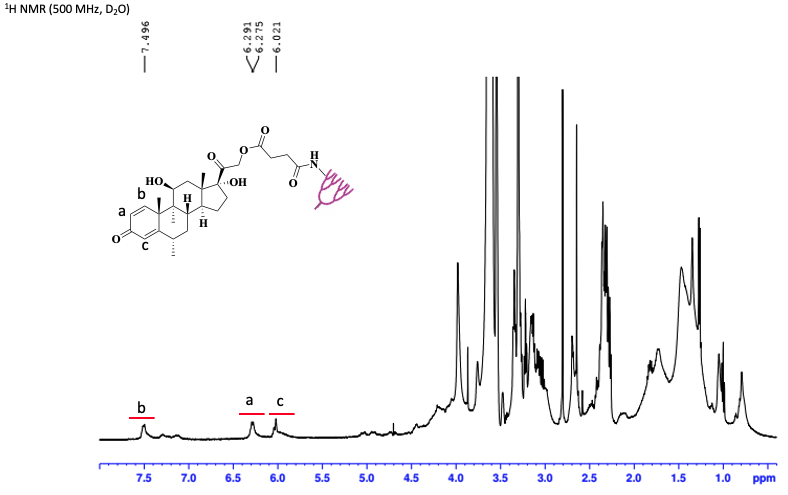


**Figure S24.** ^1^HNMR spectrum of methylprednisolone conjugated with G2HP BPL molecules in DMSO-d_6_.


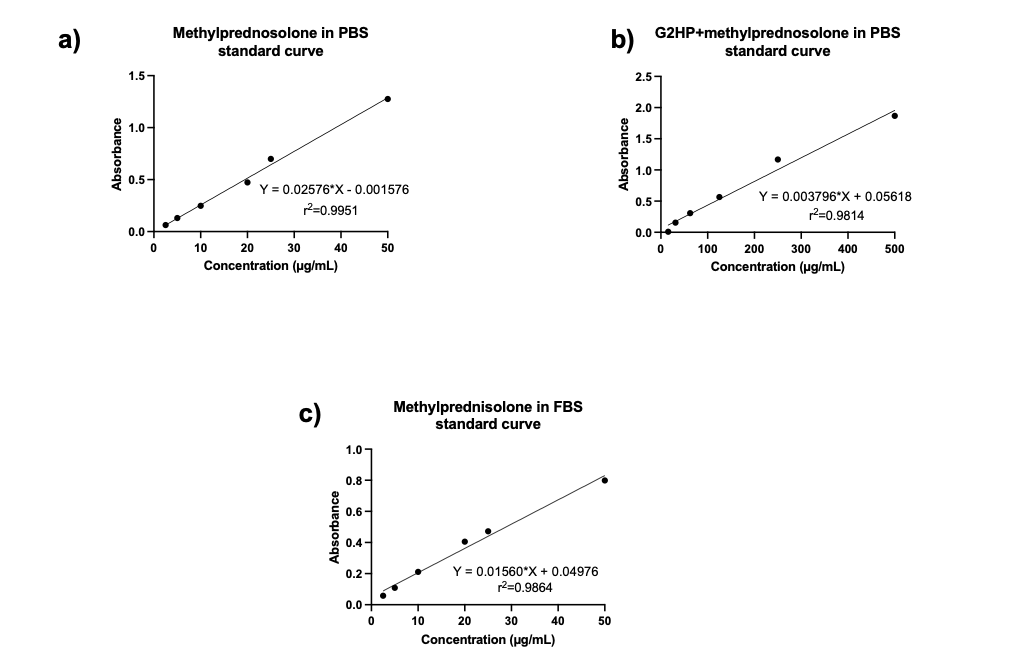


**Figure S25.** Standard calibration curves for (a) methylprednisolone and (b) G2HP conjugated with methylprednisolone in PBS at concentrations of 2.5-50 µg/mL and 15.625-500 µg/mL respectively obtained via UV/Vis absorbance spectroscopy (λ_max_ ~255 nm). (c) standard calibration curve for methylprednisolone in PBS with 10% FBS. At concentrations of 2.5-50 µg/mL obtained via UV/Vis absorbance spectroscopy (λ_max_ ~260 nm).

**Table S17.** Two-way ANOVA with Bonferroni’s post-hoc test from methylprednisolone release from G2HP BPL molecules in PBS and PBS in 10% FBS over 3 days (Figure 7d).

| Source of Variation | % of total variation | P value | P value summary |
| --- | --- | --- | --- |
| Time x Solvent | 8.532 | 0.0004 | *** |
| Time | 18.51 | <0.0001 | **** |
| Solvent | 41.34 | 0.0794 | ns |
| Replicate | 30.20 | <0.0001 | **** |

**Table S18.** Multiple comparisons from two-way ANOVA with Bonferroni’s post-hoc test from methylprednisolone release from G2HP BPL in PBS and PBS in 10% FBS over 3 days (Figure 7d).

| Bonferroni's multiple comparisons test | Mean Diff. | 95.00% CI of diff. | Summary | Adjusted P Value |
| --- | --- | --- | --- | --- |
| Comparison of release in PBS and PBS with 10% FBS at different times | | | | |
| Day 1 |  |  |  |  |
| PBS vs. PBS with 10% FBS | -9.667 | -26.07 to 6.740 | ns | 0.2235 |
| Day 2 |  |  |  |  |
| PBS vs. PBS with 10% FBS | -14.00 | -30.41 to 2.407 | ns | 0.0877 |
| Day 3 |  |  |  |  |
| PBS vs. PBS with 10% FBS | -28.00 | -44.41 to -11.59 | ** | 0.0029 |
| Comparison of release in PBS and 105 FBS over time | | | | |
| PBS |  |  |  |  |
| Day 1 vs. Day 2 | -2.333 | -8.224 to 3.557 | ns | 0.7993 |
| Day 1 vs. Day 3 | -4.667 | -10.56 to 1.224 | ns | 0.1317 |
| Day 2 vs. Day 3 | -2.333 | -8.224 to 3.557 | ns | 0.7993 |
| Comparison of release in PBS and 105 FBS over time | | | | |
| PBS with 10% FBS |  |  |  |  |
| Day 1 vs. Day 2 | -6.667 | -12.56 to -0.7764 | * | 0.0275 |
| Day 1 vs. Day 3 | -23.00 | -28.89 to -17.11 | **** | <0.0001 |
| Day 2 vs. Day 3 | -16.33 | -22.22 to -10.44 | **** | <0.0001 |


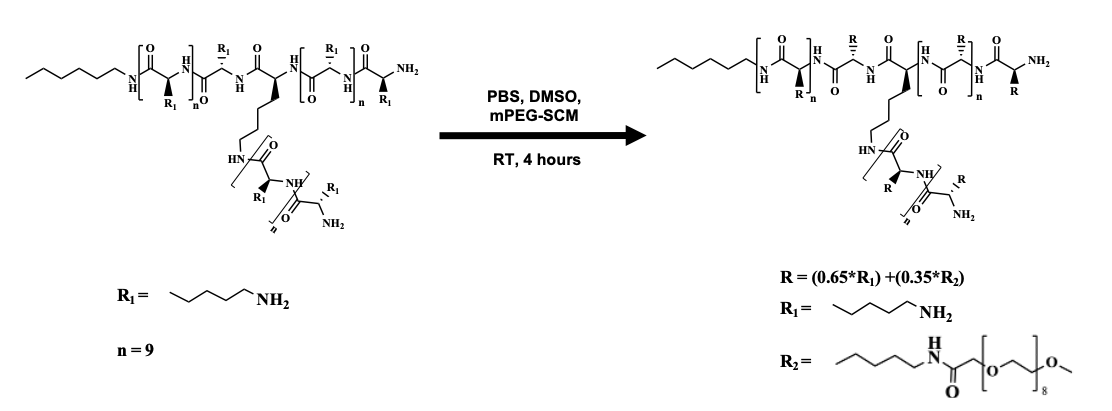


**Figure S26.** PEGylation of high PEGylation (HP) BPL molecules.
